# Engineered B cells expressing an anti-HIV antibody enable memory retention, isotype switching and clonal expansion

**DOI:** 10.1038/s41467-020-19649-1

**Published:** 2020-11-17

**Authors:** Alessio D. Nahmad, Yuval Raviv, Miriam Horovitz-Fried, Ilan Sofer, Tal Akriv, Daniel Nataf, Iris Dotan, Yaron Carmi, David Burstein, Yariv Wine, Itai Benhar, Adi Barzel

**Affiliations:** 1 The School of Neurobiology, Biochemistry and Biophysics, The George S. Wise Faculty of Life Sciences, Tel Aviv University, 69978 Tel Aviv, Israel; 2 Department of Pathology, The Sackler School of Medicine, Tel Aviv University, 69978 Tel Aviv, Israel; 3 The School of Molecular Cell Biology and Biotechnology, The George S. Wise Faculty of Life Sciences, Tel Aviv University, 69978 Tel Aviv, Israel

**Keywords:** Adaptive immunity, Immunotherapy

## Abstract

HIV viremia can be controlled by chronic antiretroviral therapy. As a potentially single-shot alternative, B cells engineered by CRISPR/Cas9 to express anti-HIV broadly neutralizing antibodies (bNAbs) are capable of secreting high antibody titers. Here, we show that, upon immunization of mice, adoptively transferred engineered B cells home to germinal centers (GC) where they predominate over the endogenous response and differentiate into memory and plasma cells while undergoing class switch recombination (CSR). Immunization with a high affinity antigen increases accumulation in GCs and CSR rates. Boost immunization increases the rate of engineered B cells in GCs and antibody secretion, indicating memory retention. Finally, antibody sequences of engineered B cells in the spleen show patterns of clonal selection. Therefore, B cells can be engineered into what could be a living and evolving drug.

## Abstract (web summary)

Chronic antiretroviral therapy does not eradicate HIV infection. Here, the authors describe a potentially one-shot alternative by engineering B cells to express anti-HIV antibodies and undergo memory retention, isotype switching and clonal expansion

## Introduction

Chronic antiretroviral therapy does not eradicate HIV infection. Broadly neutralizing antibodies (bNAbs) can suppress viremia^1^, but they would have to be chronically administered at a higher cost. Alternatively, bNAbs can be constitutively expressed from muscle^2,3^, but antidrug antibodies (ADA) are often developed^4^, possibly due to improper glycosylation. In addition, antibodies expressed from muscle undergo neither class switch recombination (CSR) nor affinity maturation, which are necessary for long term control over diverse and continuously evolving HIV infections. These challenges might be overcome by B cell engineering. A therapeutically relevant protocol was first accomplished by lentiviral transduction of human CD34^+^ cells followed by in vitro differentiation^5^. Lentiviral transduction of mature B cells allowed the developmentally regulated expression of the membranal and secreted antibody isoforms^6^. More recently, efficient CRISPR/Cas9-mediated integration of antibody genes was demonstrated into the Ig loci of primary human B cells^7^. Integration of an antibody’s variable heavy chain into the immunoglobulin heavy (IgH) locus further allows somatic hypermutation (SHM) and class switch recombination in vitro, when the endogenous constant segments are utilized using appropriate splicing signals^8^. In immunocompetent mice, adoptive transfer of B cells, engineered to express HIV-bNAbs, facilitated the production of HIV-neutralizing antibody titers^9^. Integration of single-chain anti-RSV antibodies into the IgH locus further allowed protection from infection^10^. However, in all previous in vivo studies neither immunological memory nor clonal selection was demonstrated, significantly hindering clinical application of the technology against the highly diverse and rapidly evolving HIV.

In this work, we overcome these limitations by combining Toll-like receptor (TLR)-mediated ex vivo activation with in vivo prime-boost immunization. We demonstrate that engineered B cells enable immunological memory and clonal selection that may contribute to addressing viral variability between patients and to counteracting viral escape.

## Results

### B cells can be engineered to express bNAbs using CRISPR/Cas9 and AAV

**Fig. 1:**
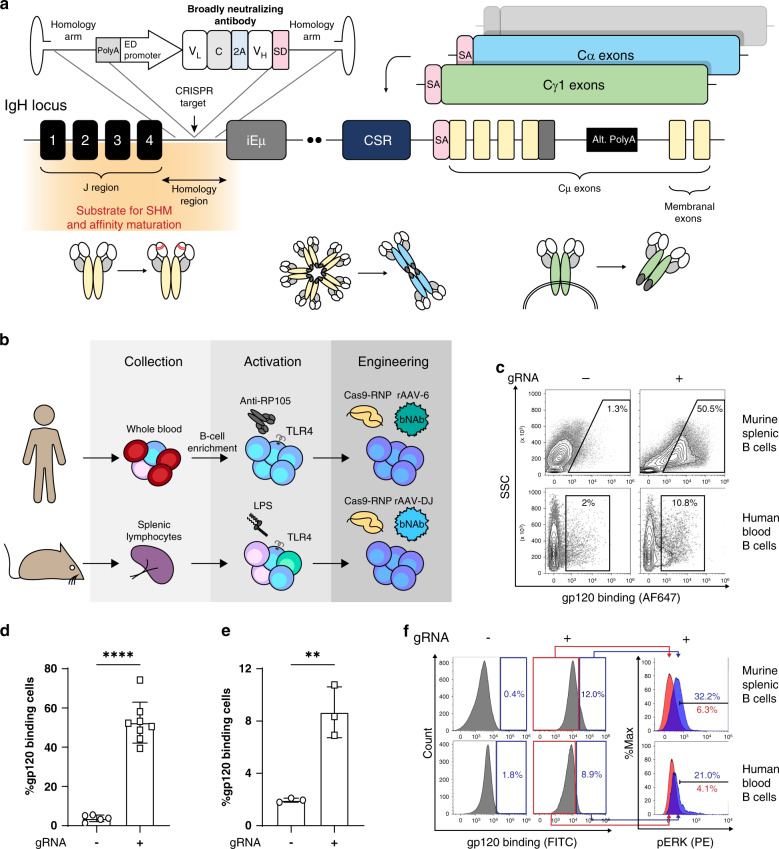
Engineering B cells to express an anti-HIV bNAb. **a** Targeting scheme. An rAAV-delivered cassette is targeted to the J-C intron of the IgH locus using CRISPR/Cas9. The bicistronic cassette encodes the light and heavy chains of the 3BNC117 anti-HIV bNAb, under the control of an enhancer dependent (ED) promoter. Splicing with endogenous constant segments allows the expression of a BCR and differentiation into memory B cells and Ig secreting plasma cells upon subsequent antigen-induced activation and alternative polyadenylation (Alt. PolyA). Targeting the J-C intron upstream of the intronic enhancer (iEμ) and switch region further facilitates CSR and SHM. See also Supplementary Fig. 1. **b** Activation and engineering scheme. Human B cells are collected from blood samples, activated using an anti-RP105 (TLR4 homolog) antibody, electroporated by CRISPR/Cas9 RNP and transduced using rAAV-6. Splenic B cells are activated using the TLR4 agonist LPS electroporated by CRISPR/Cas9 RNP and transduced using rAAV-DJ. **c** Flow cytometry plots measuring binding of the HIV gp120 antigen by the 3BNC117 BCR following activation and engineering of primary cells. Cells transduced with the donor rAAV and without gRNA serve as a negative control, gating on live, singlets. **d**, **e** Quantification of C for mouse (*n* = 5 for –gRNA and *n* = 8 for +gRNA, each dot represents a biologically independent sample, data represented as mean values +/− SD) **d** and human (*n* = 3, each dot represents a biologically independent sample, data represented as mean values +/− SD) **e** primary cells. ****p < 0.0001, **p = 0.0040; two-tailed *t*-test. **f** Flow cytometry plots demonstrating ERK phosphorylation in primary mouse or human B cells engineered with 3BNC117 and in vitro activated with the gp120 antigen of the YU2.DG HIV strain, gating on singlets.

We use CRISPR/Cas9 and recombinant adeno associated viral vectors (rAAV) to target the integration of the 3BNC117^11^ HIV-bNAb under an enhancer dependent (ED) Ig promoter^12^ into the J-C intron of the IgH locus (Fig. 1a and Supplementary Fig. 1). We chose 3BNC117, a potent CD4-mimic HIV-bNAb, because, in combination with the 10-1074 bNAb, it was recently shown to induce viral suppression in viremic individuals^13^ and in individuals undergoing treatment interruption^14^. Our bi-cistronic bNAb cassette encodes the full light chain and the variable segment of the heavy chain (VH) of 3BNC117 separated by a furin cleavage site and a 2A-peptide for ribosomal skipping. The VH is followed by a splice donor sequence to allow fusion to constant segments and initial expression of the bNAb as a membranal B cell receptor (BCR). Our design may allow disruption of the endogenous IgH chain while facilitating antigen-induced activation of engineered B cells upon immunization, leading to differentiation into memory and plasma cells, as well as to CSR, SHM, and affinity maturation (Fig. 1a and Supplementary Fig. 1).

First, CRISPR/Cas9 gRNAs were designed to promote cleavage of the mouse and the human IgH J-C introns. We chose the specific target positions to allow donor–vector design with sufficiently long homology arms, that contain neither the potentially oncogenic intronic-enhancer nor sequences corresponding to genomic segments that may be deleted during VDJ recombination. CRISPR/Cas9 cleavage produced up to 33% and 21% InDels, by the T7E1 assay, at the IgH locus of an immortalized pro B cell line (ImProB)^15^ and the Ramos human B cell line, respectively (Supplementary Fig. 2A). Different gRNAs were used to disrupt the respective endogenous kappa light chains (IgK) in order to avoid chain mispairing. The IgK gRNAs target the splice acceptor junctions of the IgK constant segments, in order to avoid targeting the IgK chain of the transgenic bNAb. Following CRISPR/Cas9 cleavage, IgK expression was ablated in up to 19% and 16% in the mouse and human cell lines, respectively (Supplementary Fig. 2B). Next, we combined the CRISPR/Cas9 ribonucleoprotein (RNP) electroporation with rAAV transduction. We first used an rAAV encoding a GFP-cassette to validate that the activity of our Ig promoter variant is dependent upon on-target integration next to the Intronic enhancer. Indeed, high and stable GFP expression was demonstrated in the ImProB cell line, but only when the appropriate gRNA was co-delivered (Supplementary Fig. 2C). When using the bNAb cassette (Fig. 1a and Supplementary Fig. 1), the combined CRISPR/Cas9 electroporation and rAAV transduction resulted in 3BNC117 BCR expression in 9% and 8% of cells in the mouse and human lines, respectively (Supplementary Fig. 2D). mRNA analysis confirmed splicing of the integrated 3BNC117 with the expected constant segment in each cell line (Supplementary Fig. 2E).

### Engineered B cells can be activated by HIV antigens in vitro

**Fig. 2:**
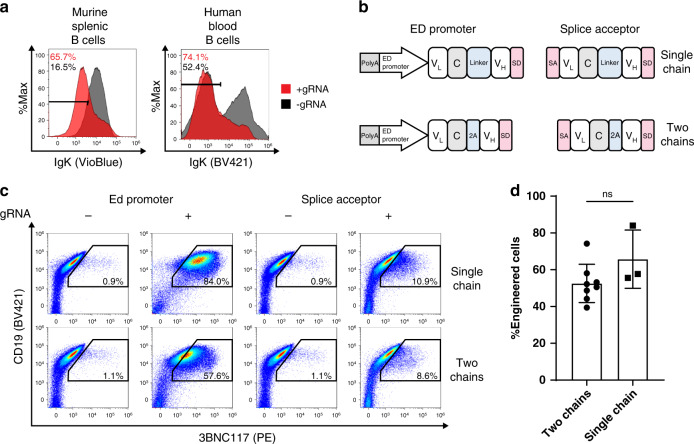
Engineering B cells with a reduced risk for the mispairing of transgenic and endogenous chains. **a** Flow cytometry of gRNA-dependent IgK ablation, gating on live, singlets for human cells and live, singlets, CD19^+^ for mouse cells. **b** Scheme of 4 alternative rAAV donor vectors where the 3BNC117 bNAb is coded as two chains separated by a furin cleavage site and a sequence coding for a 2A peptide (bottom) or where the bNAb is coded as a single chain using a linker (up) and is preceded by an ED promoter (left) or a splice acceptor sequence (right). **c** Representative analysis by flow cytometry of 3BNC117 expression in primary mouse splenic lymphocytes monitored two days following transduction and detected by anti-idiotypic antibody. Gating on live, singlets. **d** Quantification of **c** for ED promoter driven cassettes, two-tailed *t*-test (*n* = 7 for the two chain and *n* = 3 for the single chain construct, each dot represents a biologically independent sample, data represented as mean values +/− SD).

Efficient engineering of primary B cells may require activation through either the CD40 pathway^8,10^ or the TLR pathway^9^. CD40 ligation can produce germinal center (GC)-like B cells and antibody secreting plasma cells ex vivo^16^. However, B cells activated ex vivo through CD40 ligation express high levels of CD80, which are associated with a reduced propensity for further activation in vivo upon immunization^17,18^. In particular, these cells have a diminished capacity to home to GCs, establish immunological memory and undergo SHM and affinity maturation^10,18^. In contrast, TLR-pathway mediated activation of donor B cells, coming from a transgenic mouse, was recently shown to allow homing to recipient’s GCs upon immunization^9^. We therefore activated mouse splenic B cells using the TLR4 agonist lipopolysaccharide (LPS) and activated human blood B cells using an antibody against the TLR4 homolog RP105 (Fig. 1b). CRISPR/Cas9 RNP electroporation led to 30% and 29% InDel formation at the mouse and human IgH loci, respectively (Supplementary Fig. 3A). Following a subsequent rAAV transduction, up to 74% and 11% of cells expressed the 3BNC117 BCR amongst the activated mouse and human cells, respectively (Fig. 1c–e). Correct integration was validated by Sanger sequencing (Supplementary Fig. 3B). The distributions of phenotypes and isotypes were similar among 3BNC117 expressing and nonexpressing cells (Supplementary Fig. 3C, D). Importantly, as we activated the cells through the TLR rather than CD40 pathway, the engineered cells express low levels of CD80 (Supplementary Fig. 3E), implying increased propensity for antigen-induced activation. 3BNC117 secretion was minor but detectable (Supplementary Fig. 3F). In addition, high viability was retained following activation, electroporation, and transduction of both mouse and human cells (Supplementary Fig. 3G–I). Engineered B cells of both species could be further activated ex vivo by incubation with the HIV gp120 antigen, leading to ERK phosphorylation (Fig. 1f). We validated that the promoter we used is active in the primary cells only upon CRISPR-dependent integration next to the intronic enhancer (Supplementary Fig. 4A). In addition, our cassette codes for a strong polyA upstream of the ED promoter (Fig. 1a) preventing aberrant splicing with the endogenous transcript (Supplementary Fig. 4B, C). Chain mispairing could in turn be avoided by co-transfection of additional gRNAs, leading to IgK ablation in 49% and 45% of mouse and human IgH alleles, respectively (Fig. 2a). Mispairings between transgenic and endogenous chains can also be reduced by coding 3BNC117 as a single chain antibody^10^ (Fig. 2b, c, top, 2D). In order to further increase safety, we demonstrated efficient B cell engineering with a promoter-less 3BNC117 cassette, allowing expression only upon integration into the IgH locus followed by splicing with the endogenous transcript (Fig. 2b, c, bottom). While the promoter-less design was associated with a reduced engineering rate, the single-chain design allowed for a high rate of engineering. Importantly, low rates of InDels, implying off target cleavage, could be detected in the only four loci in the human genome containing a sequence that differs from the IgH target site at exactly two positions (Supplementary Fig. 4D–F). A nonstatistically significant trend for a higher InDel rate was detected in a chromosome 7 lncRNA intron, being one of three sites differing from the target site at exactly three positions and chosen for analysis as they are identical to the target site at the 4 PAM-proximal positions. Notably, off-target InDel activity has previously been shown to occur at sites with up to six mismatches, and even low off-target rates may be clinically significant when infusing millions of engineered cells. Supplementary Fig. 4G, H thus reports the number of potential off-target sites for each mismatch number for the human and mouse gRNAs. Future implementations of CRISPR mediated B cell engineering in the clinical setting may further require a deep unbiased analysis of off-target cleavage distribution^19^. The ultimate cleavage site in the human IgH J-C intron can then be chosen (Supplementary Data 1) based on it being associated with a minimal off target cleavage profile.

### Engineered B cells can undergo antigen-induced activation in vivo

**Fig. 3:**
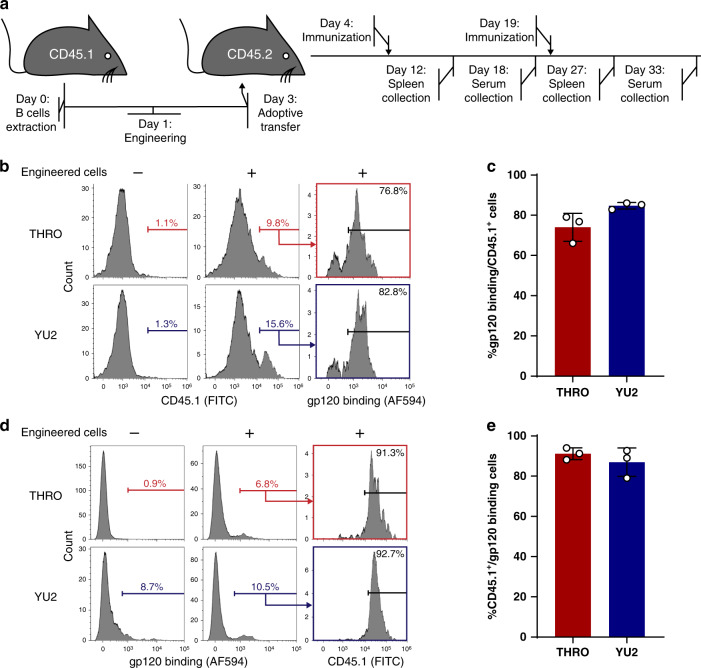
Adoptively transferred engineered B cells can undergo antigen-induced activation in-vivo. **a** Experimental scheme of the in vivo assays. Splenic B cells, from C57BL/6 CD45.1 mice, were engineered as in Fig. 1 and infused to otherwise syngeneic CD45.2 recipient mice. Different mice groups were immunized on the following day with gp120 antigens from either the THRO4156.18 (THRO) or the YU2.DG (YU2) HIV strains. When boosted by an additional injection, the mice received the same antigen as in the prime injection. Different mice groups were sacrificed 8 days following injections for spleen collection or terminally bled 14 days after injection for serum collection. **b** Representative analysis by flow cytometry of the accumulation of engineered cells in the GCs of mice immunized with either the YU2.DG or the THRO4156.18 gp120 antigens, 8 days following a prime antigen injection. Gating on live, singlets, B220^+^, GL-7^+^. **c** Quantification of **b**. Each dot represents a different mouse. Error bars represent SD (*n* = 3, represents amouse, data represented as mean values +/− SD). **d** Representative analysis by flow cytometry of CD45.1 expression among gp120 binding GC cells. Pre-gating on singlets, live, B220^+^, GL-7^+^. **e** Quantification of **d**. Each dot represents a different mouse. Error bars represent SD (*n* = 3, each dot represents a mouse, data represented as mean values +/− SD). For gating strategy see Supplementary Fig. 12.

In order to assess functionality in vivo, we first engineered activated splenic B cells of CD45.1 C57BL/6 mice to express 3BNC117, as in Fig. 1a, and then we adoptively transferred the cells into an otherwise syngeneic CD45.2 mice. Each recipient mouse received 1.5–2.2 M donor cells, such that the number of 3BNC117 expressing cells transferred was set at 112,500. Different groups of mice subsequently received only prime or both prime and boost immunizations, and were terminally bled to assess serum antibody concentration or sacrificed to analyze the splenic B cell population (Fig. 3a). Different groups were immunized with the gp120 antigen of the YU2.DG HIV strain, efficiently neutralized by 3BNC117, or by the gp120 antigen of the THRO4156.18 HIV strain, which is poorly neutralized by 3BNC117^20^. 3BNC117 has a much higher affinity to the YU2.DG antigen (Supplementary Fig. 5A), but the antigens are otherwise comparable, both belonging to the clade B HIV-1 strains. CD45.1^+^ donor cells were found in germinal centers of mice receiving adoptive transfer and subsequent immunizations with either of the antigens (Supplementary Fig. 5B). While less than 10% of the transferred cells were gp120 binders, the vast majority of CD45.1 expressing cells in the GCs bound gp120 (Fig. 3b, c), a strong indication for antigen-induced homing to GCs. Furthermore, while gp120 is immunogenic to mice, irrespective of B cell engineering (Supplementary Fig. 5C, D), 8 days following prime immunization with either antigen, relevant splenic GCs were monopolized by donor cells, with more than 90% of gp120 binding cells expressing CD45.1 (Fig. 3d, e). Therefore, in patients, activation of engineered B cells encoding neutralizing antibodies may efficiently compete with the endogenous response, which is often non-neutralizing^21,22^.

### Engineered B cells enable memory retention

**Fig. 4:**
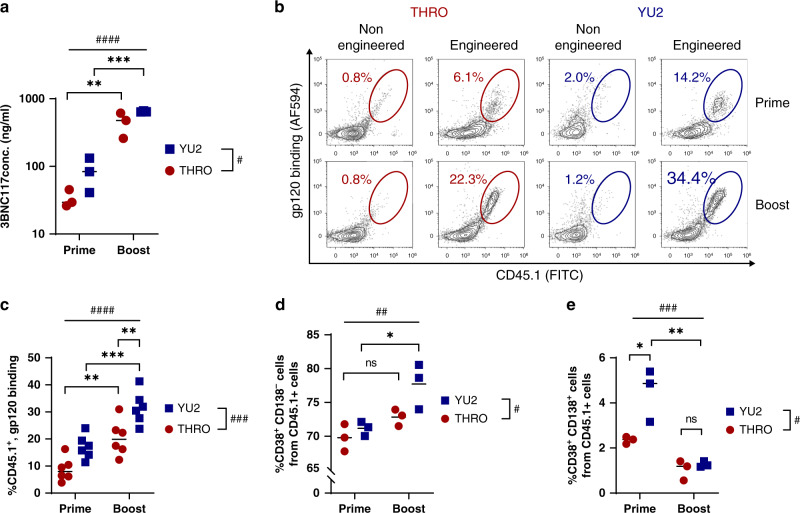
Adoptively transferred engineered B cells enable memory retention upon immunization. **a** ELISA of sera collected 14 days following either prime or boost immunization, quantified using an anti-idiotypic antibody to 3BNC117. ####pv < 0.0001, #pv = 0.0465 for two-way ANOVA and **pv = 0.0024, ***pv = 0.0003 for Tukey’s multiple comparison (*n* = 3, each dot represents a mouse). **b** Analysis by flow cytometry of CD45.1 expression and gp120 binding in the GCs of mice following prime or boost immunizations, gating on live, singlets, B220^+^, GL-7^+^. **c** Quantitation of **b**. ####pv < 0.0001, ###pv = 0.0003 for two-way ANOVA and ***pv = 0.0008 and **upper = pv = 0.0095 and **lower = pv = 0.0067 for Tukey’s multiple comparison (*n* = 6, each dot represents a mouse). **d**, **e** Analysis by flow cytometry of CD38 or CD138 expression among donor derived cells in the spleens of recipient mice after prime or boost immunizations by the gp120 antigens from either the THRO4156.18 (THRO, Red) or the YU2.DG (YU2, Blue) HIV strains, gated on live, singlets, CD45.1^+^. ###pv = 0.0003, ##pv = 0.0044, #(D) = pv = 0.0338, #(E) = pv = 0.0125, for two-way ANOVA and **pv = 0.0012, *(D) = pv = 0.0222, *(E) = pv = 0.0143, Tukey’s multiple comparison (*n* = 3, each dot represents a mouse). For gating strategy see Supplementary Fig. 12.

Fourteen days of post-immunizations with the YU2.DG gp120 antigen, we found higher serum concentrations of the 3BNC117 Ab compared to the concentrations following immunizations with the THRO4156.18 antigen (Fig. 4a). However, importantly, a log increase in serum Ab concentrations was measured 14 days following boost immunizations, reaching nearly 1μg/ml using either of the antigens (Fig. 4a, Supplementary Fig. 5E). Such high 3BNC117 serum concentrations were previously demonstrated to allow broad HIV neutralization^23^. The rate of engineered cells in the GCs has also significantly increased 8 days following boost immunization with either of the antigens (Fig. 4b, c and Supplementary Fig. 5F-G). Compared to mice receiving both prime and boost immunizations, rates of engineered cells in the GCs were lower in mice analyzed at a late time point after receiving only an early prime immunization and in mice receiving only a late prime immunization (Supplementary Fig. 6A, B). Therefore, importantly, the boost effect can be strictly attributed to the retention of immunological memory. Indeed, immunophenotyping of the donor cells in the spleen revealed that the engineered cells were differentiating into both CD38^+^ CD138^+^ Ig secreting cells and CD38^+^ CD138^−^ (which include memory B cells) (Fig. 4d, e and Supplementary Fig. 6C). Interestingly, following immunization with the YU2.DG gp120 antigen we found higher rates of CD38^+^ CD138^−^ B cell compared to following immunization with the THRO4156.18 antigen. In addition, the rate of splenic CD38^+^ CD138^−^ B cells was increased while the rate of plasma cells was decreased following boost immunization with either of the antigens, in concordance with natural mouse boost responses^24^.

### Engineered B cells undergo CSR, SHM, and clonal expansion in vivo

**Fig. 5:**
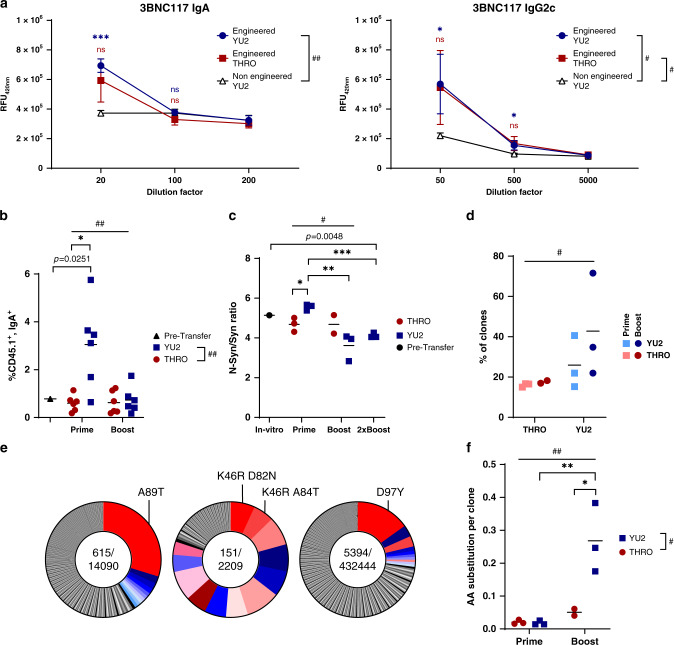
Adoptively transferred engineered B cells can undergo CSR and clonal expansion upon immunization. **a** Isotype specific anti-idiotypic ELISA measuring 3BNC117 isotypes in mice sera collected after boost immunizations. # left = pv = 0.0278, # right = pv = 0.0309, ##pv = 0.0014 for Dunnett’s multiple comparisons and ***pv = 0.0003 and * left = pv = 0.0343 and * right = pv = 0.0461 for two-tailed *t*-test. Comparisons performed to sera of mice receiving adoptive transfer of non-engineered B cells and boost immunized with the YU2.DG gp120 antigen. **b** Analysis by flow cytometry of IgA and CD45.1 expression among GC cells after prime and boost immunizations, gating on live, lymphocytes, GL-7^+^, B220^+^. ## upper = pv = 0.0087 and ##pv = 0.0041 for two-way ANOVA, *pv = 0.0074 for two-tailed *t*-test and indicated *p* value is for one-sample *t*-test **c** Ratio of non-synonymous to synonymous mutations in the different samples. #pv = 0.0143 for two-way ANOVA between the prime and boost cohorts. *pv = 0.0169, **pv = 0.0088, ***pv = 0.0002 for two-tailed *t*-test and indicated *p* value is for one-sample *t*-test. **d** Quantitation of the clonal expansion by measuring polarity: the relative cumulative share of the ten most abundant clones. # pv = 0.0496; two-way ANOVA. **e** Pie charts of mice immunized with the YU2.DG gp120 antigen and having at least one clone representing more than >10% of the mutant repertoire. The most abundant clones in each mouse were colored. Shades of red indicate clones that were not found in the ten most abundant clones of other mice (Supplementary Fig. 10). Shades of blue indicate shared clones. Indicated clones are the K46R, A89T, and D97Y. **f** Quantitation of amino acid (AA) substitutions per clone in mice immunized once or twice with the YU2.DG or THRO4156.18 gp120 antigens. ##pv = 0.005, #pv = 0.0177 for two-way ANOVA and **pv = 0.0043, *pv = 0.0167 for Tukey’s multiple comparison. For **c**, **d**, **f** *n* = 3 for all except THRO Boost samples in which *n* = 2, each dot represents a mouse.

CSR may be necessary to ensure both humoral and mucosal protection from HIV surge. Indeed, IgG_1_, IgG_2_, and IgA isotypes of the 3BNC117 bNAb were found in the sera of treated mice in addition to the IgM isotype (Fig. 5a and Supplementary Fig. 7A–C). Class switched 3BNC117 antibodies were more prevalent in sera when the YU2.DG gp120 antigen was used for immunization, and engineered cells expressing the IgA isotype were found in the GCs of treated mice only upon prime immunization by the YU2.DG gp120 antigen (Fig. 5b). As CSR often precedes GC homing^25^, this trend is in agreement with the higher rates of GC B cells in mice immunized by the YU2.DG antigen. Notably, rates of IgA expression among donor cells in the GCs, after immunizations with YU2.DG, were higher than the pre-implantation rates, implying antigen-induced in vivo CSR (Fig. 5b and Supplementary Fig. 7D).

Finally, in order to assess in vivo SHM and clonal expansion among engineered B cells, we used a synonymously recoded 3BNC117 allele, enriched for sequence hotspots of activation-induced-cytidine-deaminase (AID, catalyzing SHM) (Supplementary Fig. 8). Accumulation of engineered B cells in the GCs (Supplementary Fig. 8C) and antibody concentrations in the serum (Supplementary Fig. 8D, E) were similar, following immunizations, whether the adoptively transferred B cells were engineered to express 3BNC117-W.T. or the recoded variant: 3BNC117-opt. We harvested RNA from the spleens of mice receiving engineered cells and amplified the bNAb *V*_H_ sequence from the cDNA (Supplementary Fig. 9A) for analysis by Illumina sequencing. We found strong evidence for clonal expansion. While some mutations were found to arise during AAV preparation and B cell engineering^26^, the distribution of mutations along the 3BNC117 sequence has shifted following adoptive transfer and immunizations in patterns implying in vivo selection (Supplementary Fig. 9B). Expectedly, enrichment for AID sequence hotspots increased the total fraction of mutant sequences (Supplementary Fig. 9C). SHM and/or in vivo selection are further implied by the decrease in the ratio of non-synonymous to synonymous mutations upon adoptive transfer followed by prime and boost immunizations with the YU2.DG gp120 antigen (Fig. 5c). Furthermore, clonal expansion following YU2.DG gp120 immunizations was evident by the marked increase in the relative share of the ten most abundant clones (Fig. 5d and Supplementary Fig. 10A), and the selection of clones was similar between mice from the same cohort (Supplementary Fig. 10B–E). In particular, the three amino acid differences found in more than 10% of the variants, in their respective mice, were D97Y in the CDR3, A89T in a FR3/CDR3 border position and K46R in the CDR2 (Fig. 5e, Supplementary Fig. 10F). Clonal expansions of the K46R and A89T substitutions are supported by their association with multiple additional substitutions (Supplementary Fig. 11A–C) in their respective mice. None of these combinations of substitutions could be found in any other mouse, including in five mice adoptively transferred by the same pool of engineered B cells. This strongly indicates de novo SHM. Clonal expansion of both the K46R and the A89T substitutions is further supported by the presence of the respective R and T amino acids in the sequence of the related VRC01 and NIH45-46 antibodies^27^ (Supplementary Fig. 10F). Interestingly, immunizations with the higher affinity antigen YU2.DG, as well as boost immunizations, triggered higher accumulation of amino acid substitutions (Fig. 5f). The limited clonal expansion following immunizations with the THRO4156.18 gp120 antigen may imply that multiple or rare substitutions in the 3BNC117 sequence are necessary in order to allow sufficient improvement in the affinity toward this antigen.

## Discussion

In summary, we have demonstrated that B cells engineered to express bNAbs can undergo antigen-induced activation in mice followed by memory retention, CSR, SHM, and clonal expansion. This conclusion is corroborated by a parallel study independently demonstrating that engineered B cells could be used as a durable and evolving strategy to protect against HIV^28^. In the human setting, bNAb SHM may facilitate affinity maturation to counteract HIV diversity and high mutation rate. Moreover, patient or donor B cells may be engineered to express two or more bNAbs^14^ targeting different viral epitopes, to further diminish the risk of escape. Both safety and efficiency may be increased by expressing the bNAb as a single chain and by using promoter-less constructs that more strictly prevent expression from off target integration^29^. The therapeutic potential of our approach may best be assessed in nonhuman primates with HIV-like infections. Finally, B cell engineering as a platform technology may be applied in the future to diverse persistent infections as well as to the treatment of congenital disorders, autoimmune diseases and cancer.

## Methods

### crRNA and sgRNA for SpCas9 cleavage

A list of gRNAs used in this manuscript is presented in Supplementary Table 1. A list of alternative spCas9 cleavage sites for the mouse J-C IgH intron are present in file Supplementary Data 1.

### Plasmid cloning

For the mouse donor plasmid, two intronic sequences directly adjacent to the mouse target gRNA (homology arms) were PCR amplified from the ImProB cell line. Fragments were inserted by In-Fusion assembly into pAB270^29^ cut with NheI and SpeI. Finally, we inserted the sequence of the 3BNC117-W.T. donor or the sequence of the the 3BNC117-opt donor in the XhoI restriction site by Gibson Assembly (E2611, NEB).

For the human donor plasmid, the intronic sequences were amplified and Gibson Assembled into pAB270 NheI-SpeI restricted. Sequence of the human 3BNC117 donor DNA was integrated at the XhoI restriction site.

For the promotor-less donors, we amplified from C57/BL6 genomic DNA the IgM splice acceptor and replaced the PolyA-ED promoter cassette with the resulting product directly followed by a Furin-GSG-P2A cassette.

For the single chain donors, we replaced the Furin-GSG-P2A between the two chains coding sequences with a variant of a previously described sequence^30^ (Supplementary Data 2).

### rAAV production

rAAV-DJ and rAAV-6 donors were produced in 293t cells (ATCC CRL-3216) by transient transfection. In short, 10–14 15 cm dishes were transfected when cells were 80% confluent pAd5 (helper plasmid), rAAV-DJ or rAAV-6 genome plasmid and Donor plasmid at a 3:1:1 ratio in Polyethylenimine (PEI) (24765-1 Polysciences Inc.). In total each plate was transfected with 41,250 ng of DNA. Purification was performed with the AAVpro Extraction Kit (6666, Takara) according to the manufacturer’s protocol, followed by titer quantification using qPCR with SYBRGreen (4309155, ThermoFisher or PB20.12 PCRBiosystems).

### Cell lines

For cell lines, ImProB^15^, A-20^31^, Ramos, and i.29 IgA^32^ electroporations were performed with 18.3pmol Cas9 and 22pmol gRNA, at 1.0E5cells/μl in OptiMEM (31985-047, gibco) using 10 μl tips in a Neon electroporation system (Invitrogen). For the human cell line, parameters were: 1350 v 30 ms 1 pulse and for the mouse cell lines: 1600v 20 ms, 1 pulse. All cell lines were grown in 1640 RPMI (01-100-1A, Biological Industries) supplemented with 10% HI FBS (04-127-1A, Biological Industries), 50 μM β-Mercaptoethanol and P/S (03-031-1B, Biological Industries). Transductions were performed with a 50,000 MOI of rAAV-DJ for mouse cell lines, and a 130,000 MOI of rAAV-DJ for human cell line. Efficiency of editing was determined 3 days following electroporation.

For plasmid electroporations we used 3 µg plasmid DNA/1E06 cells/10 µl Neon tip.

The Ramos cell line (ATCC CRL-1596) was provided by the Benhar lab, Tel Aviv University. The i.29 cell line was provided by the Zan Bar lab, Tel Aviv University. The ImProB cell line was provided by the Deriano lab, Pasteur Institute. The A-20 cell line was provided commercially (ATCC TIB-208).

### Primary human cultures

For human cells, whole blood was obtained with donor consent from the Israeli blood bank (Magen David Adom, Sheiba Medical Center) in accordance with Tel Aviv University Review Board, and PBMCs were extracted using Lymphocyte Separation Medium (0850494-CF, mpbio). Remnants of red blood cells were lysed using Red Blood Cell lysis (420301, Biolegend). B cells were enriched using the negative selection Easysep human B cell isolation kit (17954, Stemcell) and plated in 1640 RPMI (01-100-1A, Biological Industries) supplemented with 10% HI FBS (04-127-1 A, Biological Industries), 50 μM β-Mercaptoethanol, P/S (03-031-1B, Biological Industries), 2 μg/ml RP105 (312907, Biolegend) and 10 ng/ml IL7 (200-07, Peprotech).

For human primary cells, electroporation parameters were: 1750 v 20 ms 1 pulse in a Neon electroporation system (Invitrogen) at 4.0E5 cells/μl in buffer R using 10 μl tips. For RNP, Cas9 (1081059, IDT) and gRNA (IDT): complexes assemblies were generated 20 min prior to transfection with 18.3 pmol Cas9 and 66 pmol gRNA per 1E06 cells. Transductions were performed no later than 5 min following electroporation with a 10,000 MOI of rAAV-6. Efficiency of editing was determined 2 days following electroporation.

### Primary mouse cultures

For mouse cells, whole spleens were extracted from donor (CD45.1) mice and were mechanically crushed in PBS to be filtered in a 70 μm Cell Strainer (Corning). Following Red Blood Cell lysis (420301, Biolegend), cells were plated at 3.0E6 cells/ml in 1640 RPMI (01-100-1A, Biological Industries) supplemented with 10% HI FBS (04-127-1A, Biological Industries), 50 μM β-Mercaptoethanol, P/S (03-031-1B, Biological Industries), 10 μg/ml LPS (sc-3535, SantaCruz Biotechnology) and 10 ng/ml IL4 (214-14, Peprotech) was added immediately after extraction.

Cells were cultured 16–24 h and washed in PBS before transfections and plated in the same activation medium without P/S for 8–16 h following electroporations. Parameters were 1350 v 30 ms 1 pulse at 4.0E5 cells/μl in buffer R for 10 μl tips. For RNP: Cas9 (1081059, IDT) and gRNA (IDT) complexes assemblies were generated 20 min prior of transfection with 18.3 pmol Cas9 and 38 pmol gRNA per 1E06 cells. Transductions were performed no later than 5 min following electroporation with a 50,000 MOI of rAAV-DJ and cells were analyzed 2 days following electroporation. For co-culture with CD40LB feeders, splenic lymphocytes were seeded as previously described^17^.

### Flow cytometry

For samples from recipient (CD45.2) mice, cells were stained immediately following extraction. Samples were washed and resuspended in Cell Staining Buffer (420201, Biolegend). Antibodies were added and incubated for 20 min in the dark at room temperature for binding. Samples were then washed and resuspended in staining buffer before reading in an Attune NxT (Life technologies) flow cytometer. For indirect flow cytometry: samples were incubated for 5 min with 1 µg of the YU2.DG gp120 antigen in 100 µl, washed and conjugated antibodies were added in 100 µl for 15 min followed by additional washing and acquisition.

For assessing ERK phosphorylation, cells were incubated for 3 min at 37 °C in 100 µl PBS before supplementing with 5 µg of the YU2.DG gp120 antigen, incubated for one more minute, immediately put on ice and diluted by a factor of 10 with ice-cold 1:1 MetOH:Acetone and incubated for 20 min at −20 °C. Cells were then washed and stained as described above.

For viability staining, Propidium Iodide (00-6990-50, eBioscience) was added immediately before acquisition.

For assessment of IgK ablation, we used (Eq. (1)).

1 $${\mathrm{IgK}}\,{\mathrm{ablation = IgK}}^{\mathrm{ + }}_{{\mathrm{nogRNA}}} - {\mathrm{IgK}}^{\mathrm{ + }}_{{\mathrm{gRNA}}}$$

ERK phosphorylation was assessed as previously described^33^. In short, cells were washed and resuspended in PBS at 2 million cells/ml. Cells were incubated for 3 min at 37 °C before supplementing by 5 µg of the YU2.DG gp120 antigen, incubated for one more minute and immediately put on ice and diluted by a factor of 2 with MetOH and incubated for 15 min at −20 °C before staining.

For all antibodies, staining was performed at a 1:100 dilution. Pre-gating for all experiments can be found in Supplementary Fig. 12. Data were compiled and analyzed using Kaluza Analysis 2.1 (Beckman Coulter). A list of antibodies used can be found in Supplementary Table 2.

### ELISA

High binding microplates (greiner bio-one) were coated with 5 μg/ml of an anti-idiotipic antibody against 3BNC117 or 2 μg/ml of the YU2.DG gp120 antigen in PBS overnight at 4 °C. Plates were washed with PBST and blocked for two hours with 5% BSA in PBST and washed again. Plates were then applied with detection antibodies, antimouse IgA(abcam) or antimouse IgG or antimouse IgG1 or antimouse IgM (Jackson ImmunoResearch) at 2 μg/ml in PBST and were incubated for 2 h. A list of antibodies used can be found in Supplementary Table 2. Before detection with QuantaBlu (15169, ThermoFisher) according to manufacturer protocols, plates were washed for an additional round. Detection was done in a Synergy M1 Plate reader (Biotek). When absolute quantitation is presented, the concentration of 3BNC117 was determined by reference to the dilution factor of the standard curve.

gp120 Env proteins from THRO4156 clone 18 SVPB15 (data accessed from GenBankAY835448) were cloned in pcDNA3.1. The YU2.DG gp120 vector was previously described^34^ (for expression under the human CD5 leader sequence, and with a His-tag (6×) on the C terminus. Plasmids encoding gp120 were transfected into Expi293F cells (ThermoFisher A14635, provided by the Wine lab, Tel Aviv University) at a density of 2E6 cells/ml in Expi293 Expression Medium (ThermoFisher) using ExpiFectamine (ThermoFisher) according to manufacturer protocols. Supernatants were collected 7 days later and bound with Ni-NTA Agarose (30210, Qiagen) in 20 mM sodium phosphate, 0.5 M NaCl, 10 mM imidazole (ID). Beads were washed twice with the same buffer before mounting on gravity-flow polypropylene columns (Biorad). Chromatography elution was performed in three fractions: 50, 100, and 200 mM ID. Elutes were buffer exchanged to PBS using Amicon Ultra-15 Centrifugal Filter Units (Merck) following filtration in 0.22 μm filter unit (Millex).

For 3BNC117 and anti-idiotypic antibodies, 7.5 μg of a plasmid encoding the light chain and 22.5 µg of plasmid encoding heavy chain were co-transfected into Expi293F cells at a density of 2.0E6 cells/ml in Expi293 Expression Medium (A1435103, ThermoFisher) using ExpiFectamine (A14525, ThermoFisher) according to manufacturer protocols. Purification of the supernatant, 7 days post-transfection, was performed using MabSelect (GE Healthcare) following manufacturer recommendations.

### Mouse experiments

All mouse experiments were done with approval of Tel Aviv University Committee for the Use and Treatment of Laboratory Animals. 6–10-weeks-old female CD45.1 or CD45.2 C57BL/6JOlaHsd mice were housed and kept at ambient temperature of 19–23 °C, humidity of 45–65% and with a 12 h light/12 h dark cycle.

Engineered splenic B cells were transferred to recipient (CD45.2) mice (Envigo) by retro-orbital injections at 1.5–2.2 M cells/mouse in 100 μl Mg^+2^ and Ca^+2^ supplemented PBS with 5% Horse Serum. Mice were anesthetized with 0.1 mg/g Ketamine and 0.01 mg/g Xylazine prior to transfer. Number of cells to be transferred was set to be 112,500 gp120 binding cells and the ratio of cells was determined by flow cytometry prior to infusion. Cells were diluted in mock cells (electroporated without gRNA and then transduced) if needed and counted in a TC-20 automatic cell counter (Biorad). For immunizations, gp120 in PBS (200 µg/ml for 100 µl/20 µg/mice) was mixed at a 1:1 ratio with Alhydrogel 2% (vac-alu-250, Invitrogen) and injected intraperitoneally. Mice receiving nonengineered cells received adoptive transfer of 1.5 M cells/mouse. Mice receiving engineered cells but not immunized received PBS injections mixed at a 1:1 ratio with Alhydrogel instead.

Blood samples were collected by terminal bleeding in heparin. Sera from the different mice were distilled by repeated light centrifugation and collection of the supernatant until no erythrocytes were found.

### Nucleic acid manipulations

Reverse transcriptions were executed on total RNA extracted using QuickRNA microPrep kit (Zymo Research) from 1 to 2E06 cells 3 days following transfection with CRISPR-RNPs and AAV transduction. Cells transfected without the gRNA, but otherwise similarly treated, were used as control. 500–1500 ng of extracted RNA were used for each RT reaction using either M-MLV (M170A, Promega) or RevertAid (K1621, ThermoFisher) reverse transcriptase with oligo dT according to respective manufacturer instructions. Subsequent to RT reactions, PCR reactions were done using Maxima HotStart GreenMix at 35 cycles. A list of primers used for the amplification is listed in Supplementary Table 3.

For assessment of gRNA activity, genomic DNA was extracted using Quick DNA miniprep kit (Zymo Research) and 300–500 ng genomic DNA was amplified by PCR using PrimeSTAR MAX (R045A, Takara) for 30–35 cycles. Primers used for these reactions are listed in Supplementary Table 4. Amplicon DNA was denatured and reannealed in a thermocycler prior to cleaving by T7 Endonuclease 1 (New England Biolabs) at 37 °C in a 30 min reaction. Proteinase K was supplemented to the reaction and incubated for an additional 15 min at 37 °C. Cleavage was analyzed by agarose gel electrophoresis and quantified using Biovision (Vilber Lourmat) using a rolling ball for background subtraction. Efficiency was calculated using (Eq. (2)).

2 $${\mathrm{Cleavage}}\,{\mathrm{efficiency = }}\left( {{\mathrm{\% gene}}\,{\mathrm{modification}} = 100{\,}{\times}\left( {{\mathrm{1-}}\left( {{\mathrm{1}} -{\,} {\mathrm{fraction}}\,{\mathrm{cleaved}}} \right)^{{\mathrm{1/2}}}} \right)} \right).$$

For assessment of HDR mediated integration, PCR was performed on genomic DNA extracted 2 days following transduction. A list of primers used for the amplification is listed in Supplementary Table 5.

Agarose (Hy-labs) was supplemented to 40 mM Tris, 20 mM Acetate, 1 mM EDTA for a final concentration of 1–2%. Gels were run at 160 v for 20–30 min. DNA ladders used were either 100 bp DNA Ladder H3 RTU or 1 kb DNA Ladder RTU (GeneDireX).

To exclude splicing with the endogenous transcript, we extracted RNA from primary mouse splenocytes 2 days post-engineering. cDNA generation was performed using RevertAid Reverse Transcriptase (K1621, ThermoFisher). PCR amplification was performed for 35 cycles using HS Taq Red (PB10.23-02, PCRBIO) and primers listed in Supplementary Table 6.

Off-target analysis was performed using COSMID^35^ with default parameters and primers used for amplification are listed in Supplementary Table 7.

Cas-OFFinder^36^ was used for >3 mismatch off target analysis. Benchling was used for determination of additional gRNA cleaving sites in the human IgHJ-C locus (Supplementary Data 1). For TIDE, analysis was performed on PCR amplicons coming from either two Cas9 only electroporated cells (−gRNA) or from Cas9 and gRNA electroporated cells compared to Cas9 only electroporated cells (+gRNA).

### Illumina sequencing and analysis

Whole spleens were extracted from the mice, mechanistically crushed in PBS and subsequently filtered in a 70 μm Cell Strainer (Corning). The preparation underwent Red Blood Cell lysis (401301, Biolegend). cDNA was generated from Quick RNA Microprep (Zymo) extracted RNA of total splenic lymphocyte populations with the RevertAid reverse transcriptase (K1621, ThermoFisher) using Oligo-dT primers. For Initial PCR amplification, 3BNC117.W.T. VH fragments were amplified with the proofreading PrimeStarMAX polymerase (R045A, Takara) for 40 cycles. Amplicons were purified using AMPureXP beads (A63881, Beckman Coulter) at a 0.7:1 ratio. The subsequent 8 cycles PCR reaction was performed with the PrimeStarMax polymerase (R045A, Takara). A list of the primers used for the reactions is listed in Supplementary Table 8.

Libraries were purified using AMPureXP beads at a 0.7:1 ratio. Combined libraries were loaded at 5 pM with 25% PhiX control (FC-110-3001, Illumina). Sequencing was performed in a high-throughput MiSeq machine using either a v2 Nano reagent kit 2 × 250 bp or a v3 reagent kit 2 × 300 bp (MS-102-3003 and MS-103-1003, Illumina) at the Genomic Research Unit (GRU) at the Faculty of Life Sciences, Tel Aviv University. Raw fastq files were submitted to Fast Length Adjustment of Short Reads (FLASH)^37^ and resultant paired-end fasta files were submitted to High V-Quest for V gene sequence alignment and germ-line gene assignment provided by the international ImMunoGeneTics database (IMGT)^38^. IMGT aligned sequences were then processed by a series of quality control filters. For amino acid analyses the filters included: removal of truncated sequences, sequences containing stop codons and sequences with a similarity of less than 95% with the original 3BNC117 sequence. For both nucleic acid and amino acid sequences, we trimmed the 5′ and 3′ ends to reduce sequencing bias (Supplementary Fig. 10F). For nucleic acid analyses, we kept nonproductive sequences but removed high InDel count sequences. For polarity analyses, we summed frequency of the top ten clones among mutant sequences. For the analysis of synonymous vs. nonsynonymous mutations, we used the biopython^39^ alignment implementation of the Needleman–Wunsch algorithm to pairwise align the mutant *V*_H_ sequences with the *V*_H_ of 3BNC117-opt sequence.

For the N-Syn/Syn ratio, we used (Eq. (3)).

3 $$\frac{{{\mathrm{NSyn}}}}{{{\mathrm{Syn}}}}\,=\,\left( {\frac{{{\mathrm{Nsyn}}}}{{{\mathrm{Syn}}}} = \frac{{{\sum} {\left( {\alpha \ast \gamma } \right)} }}{{{\sum} {\left( {\beta \ast \gamma } \right)} }}} \right),$$

where *α* is the number of nonsynonymous mutations in a sequence, *γ* is the frequency of that sequence and *β* is the number of synonymous mutations in that sequence.

Clustal Omega^40^ was used for tree constructions (Supplementary Fig. 11A). Alignment for sequences was performed via SnapGene v5.0.7.

### Immunofluorescence staining

Slides were prepared as previously described^41^. In short, extracted tissues were immersed in 4% PFA and were subsequently immersed in 20% sucrose. Cryopreservation was performed in O.C.T (Scigen). Following blocking, slices were stained using APC-conjugated antimouse CD3 (100235, Biolegend), PE-conjugated antimouse/human B220 (103207, Biolegend), and FITC-conjugated antimouse CD45.1 (110705, Biolegend). A list of antibodies used can be found in Supplementary Table 2.

### Statistical analysis

Statistical analysis was performed using GraphPad Prism 8 to calculate *p*-values with two-way or three-way ANOVA or two tailed Student’s *t*-tests. One sample *t*-test was performed by setting expected value as for the in vitro controls. For ANOVA, Tukey’s or Dunnett’s multiple comparisons were performed as indicated.

### Reporting summary

Further information on research design is available in the Nature Research Reporting Summary linked to this article.

## Supplementary information

Supplementary Information

Descriptions of Additional Supplementary Files

Supplementary Dataset 1

Supplementary Dataset 2

Reporting Summary

## Data Availability

All data is available in the main text, in the Supplementary Data, Materials or in the Source Data files. The sequence for gp120 THRO.4156.18 was accessed from GenBank AY835448. Sequence data can be accessed in the SRA database under accession code PRJNA666317. The authors declare that all unique materials used are readily available from the authors upon MTA agreement. Source data are provided with this paper.

## Source data

Source Data
