# Supplementary material for: Engineered B cells expressing an anti-HIV antibody enable memory retention, isotype switching and clonal expansion
Source: Nat Commun. 2020 Nov 17;11:5851. doi: 10.1038/s41467-020-19649-1 (PMC7673991; doi:10.1038/s41467-020-19649-1)
Supplement: Supplementary file 1 — Supplementary Information [file 41467_2020_19649_MOESM1_ESM.pdf]

Engineered B cells expressing an anti-HIV antibody allow memory retention, isotype switching and clonal expansion

Alessio D. Nahmad[1], Yuval Raviv[1], Miriam Horovitz-Fried[1], Ilan Sofer[1], Tal Akriv[1], Daniel Nataf[1], Iris Dotan[1], Yaron Carmi[2], David Burstein[3], Yariv Wine[3], Itai Benhar[3], Adi Barzel[1*]

[1]The School of Neurobiology, Biochemistry and Biophysics, The George S. Wise Faculty of Life Sciences, Tel Aviv University, Tel Aviv 69978, Israel
[2] The Department of Pathology, The Sackler School of Medicine, Tel Aviv University, Tel Aviv 69978, Israel
[3]The School of Molecular Cell Biology and Biotechnology, The George S. Wise Faculty of Life Sciences, Tel Aviv University, Tel Aviv 69978, Israel

SUPPLEMENTARY FIGURES:

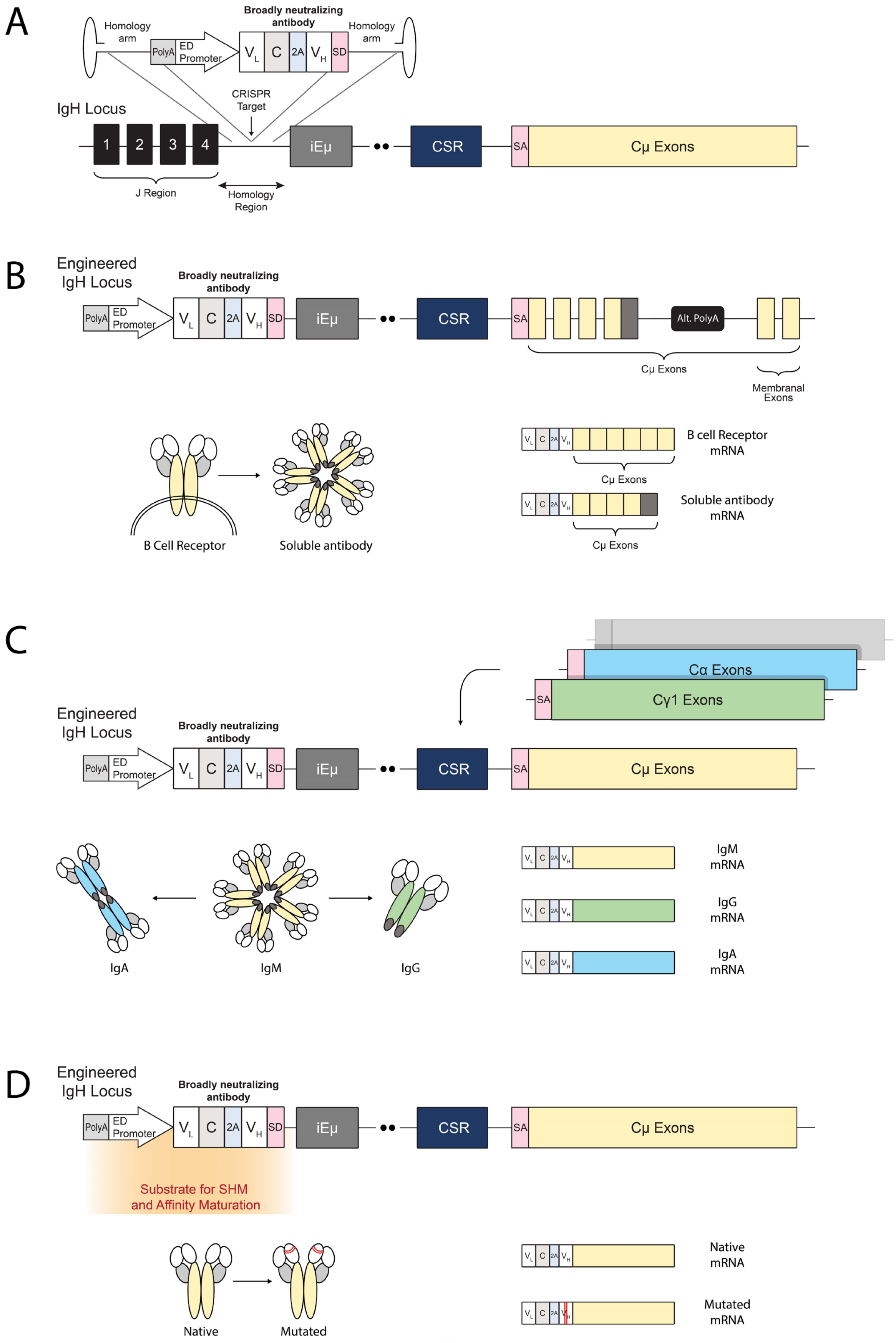
A
Broadly neutralizing antibody
Homology arm
PolyA
ED Promoter
VL C 2A VH SD
Homology arm
CRISPR Target
IgH Locus
1 2 3 4
iEμ
CSR
SA
Cμ Exons
J Region
Homology Region
B
Engineered IgH Locus
Broadly neutralizing antibody
Alt. PolyA
Cμ Exons
Membranal Exons
B Cell Receptor
Soluble antibody
B cell Receptor mRNA
Soluble antibody mRNA
C
Cα Exons
Cγ1 Exons
IgA
IgM
IgG
IgM mRNA
IgG mRNA
IgA mRNA
D
Substrate for SHM and Affinity Maturation
Native
Mutated
Native mRNA
Mutated mRNA

Supplementary Figure 1. Engineering of B cells in the IgH locus enables endogenously regulated expression of the transgenic antibody **(A)** Representative scheme of the IgH locus and CRISPR/Cas9 targeting site **(B)** Endogenously regulated alternative polyadenylation allows either inclusion or exclusion of heavy chain exons encoding for the membranal domain, leading to the expression of a B cell receptor or a secreted antibody, respectively. (C) CSR occurs downstream of the integration site. Thus, the transgenic antibody is able to undergo isotype switching. **(D)** The cassette is integrated inside a locus targeted by AID for SHM. Concordantly, the transgenic antibody undergoes endogenously regulated SHM.

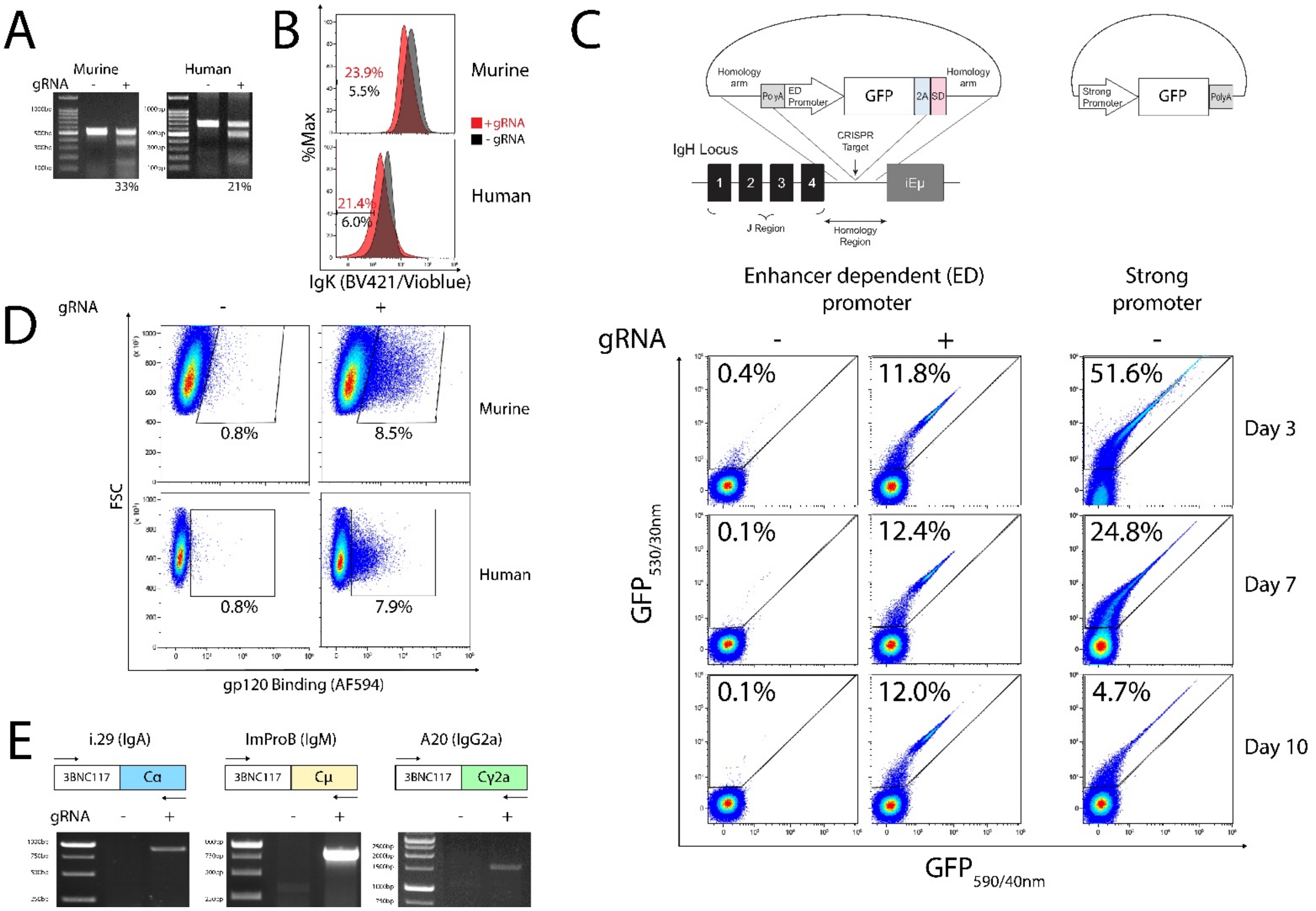

Supplementary Figure 2. (A) T7 Endonuclease 1 assay demonstrating gRNA-dependent Cas9 cleavage at the IgH J-C intron of the ImProB murine B cell line and the Ramos human B cell line. Experiments reproduced twice, independently. **(B)** Flow cytometry plots demonstrating gRNA-dependent IgK ablation in the murine and human cell lines, as above, pre-gated on live, singlets **(C)**Flow cytometry plots demonstrating CRISPR/Cas9-dependent integration of a GFP gene under an enhancer-dependent promoter (ED Promoter, left) into the IgH locus of ImProB murine cells. GFP expression was monitored at three time points after transfection. As a control, transfecting a non-integrating donor vector expressing GFP under a strong promoter (right) lead to signal dilution over time. Each row corresponds to a different time point. Gating on live, singlets. **(D)** Flow cytometry plots demonstrating gRNA-dependent 3BNC117 integration at the IgH J-C intron of the ImProB murine B cell line and the Ramos human B cell line **(E)** EtBr gels showing RT-PCR amplification products from different cell lines, each expressing a different constant domain. Amplification indicates the specific splicing between the transgene, 3BNC117, and the endogenous constant. The arrows indicate primer locations. For the ImProB cell line, experiment

reproduced twice, independently. For the i.29 cell line, experiment reproduced two times, independently. For the A20 cell line, experiment reproduced once.

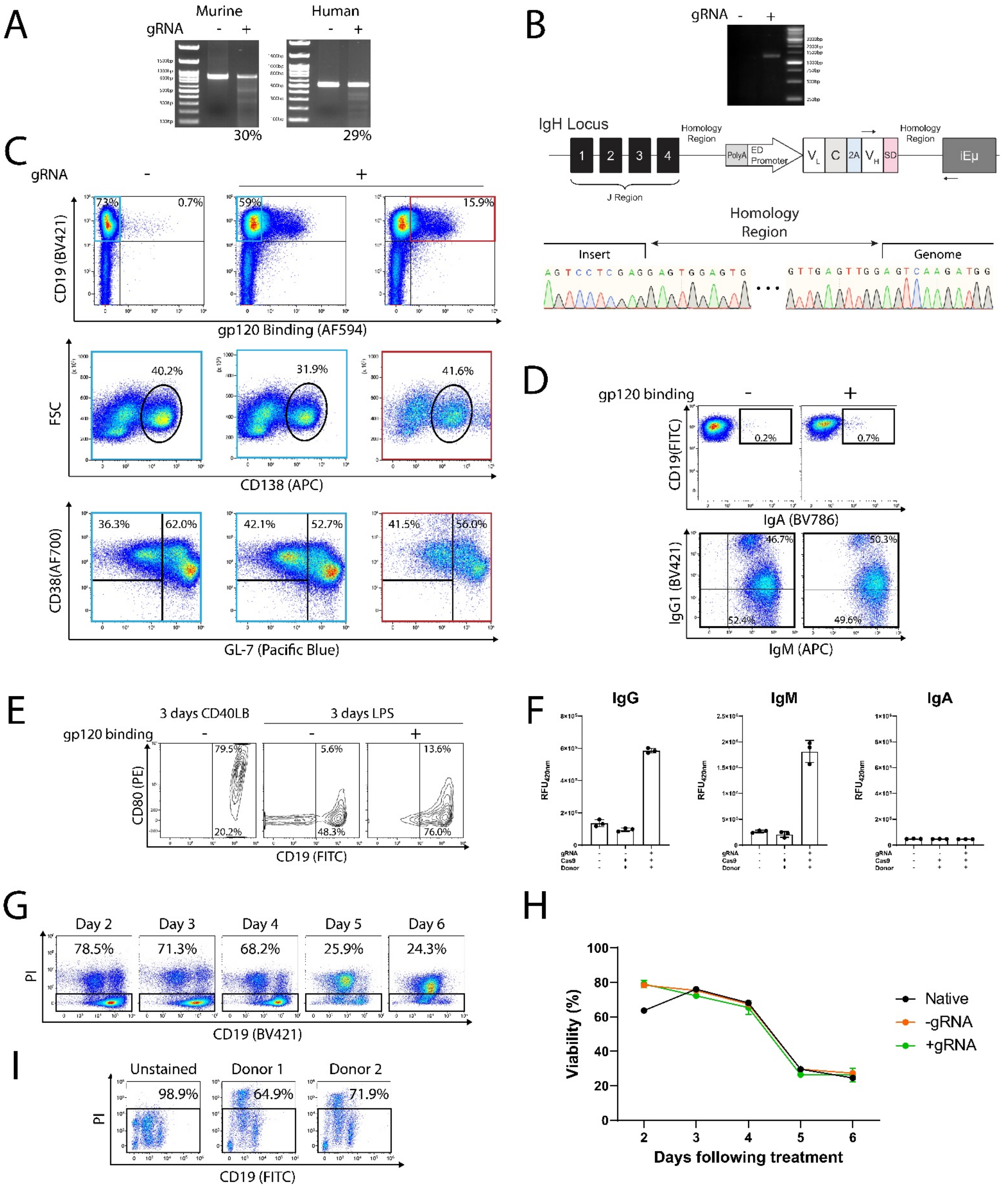

5

Supplementary Figure 3. Engineering of primary B cells can be facilitated by activation through the TLR pathway **(A)** gRNA-dependent InDel formation at the IgH loci monitored by the T7 endonuclease 1 assay. For the murine cells, the experiment was reproduced 4 times, independently. For the human cells, the experiment was reproduced once, independently. **(B)** Top: EtBr gel of a PCR amplicon demonstrating on-target integration of the 3BNC117 gene. Middle: Amplification scheme, primers are depicted as arrows. Bottom: annotated chromatogram of the Sanger sequencing. The experiment was reproduced once, independently. **(C)** Frequency of phenotypes among engineered (red) and non-engineered (blue) splenic lymphocytes, from cells transfected (+) or not transfected (-) by gRNA. For CD138 and CD38/GL-7 plots, the gating scheme is depicted by the respective colors. Pre-gating on live, singlets. **(D)** Frequency of isotypes among engineered (right) and non-engineered (left) splenic lymphocytes. Gating on live, singlets, CD19+ and gp120 non-binding (-) or gp120 binding (+). **(E)** Analysis by flow cytometry of CD80 expression in primary murine splenic lymphocytes following activation using CD40LB feeder cells or using LPS **(F)** ELISA on supernatants collected from cultures of murine splenic lymphocytes,3 days after engineering (n=3, each dot represents a technical replicate from a single experiment, data represented as mean values +/-SD). **(G)** Flow Cytometry example of murine splenic B cells following RNP transfection and AAV transduction, monitored by Propidium Iodide, up to 6 days following treatment. No pre-gating was used. **(H)** Quantification of G (n=3, data represented as the mean value of a biologically independent sample, +/-SD). **(I)** Flow Cytometry of primary human B cells 2 days following RNP Transfection and AAV transduction, monitored by Propidium Iodide, from two different donors.

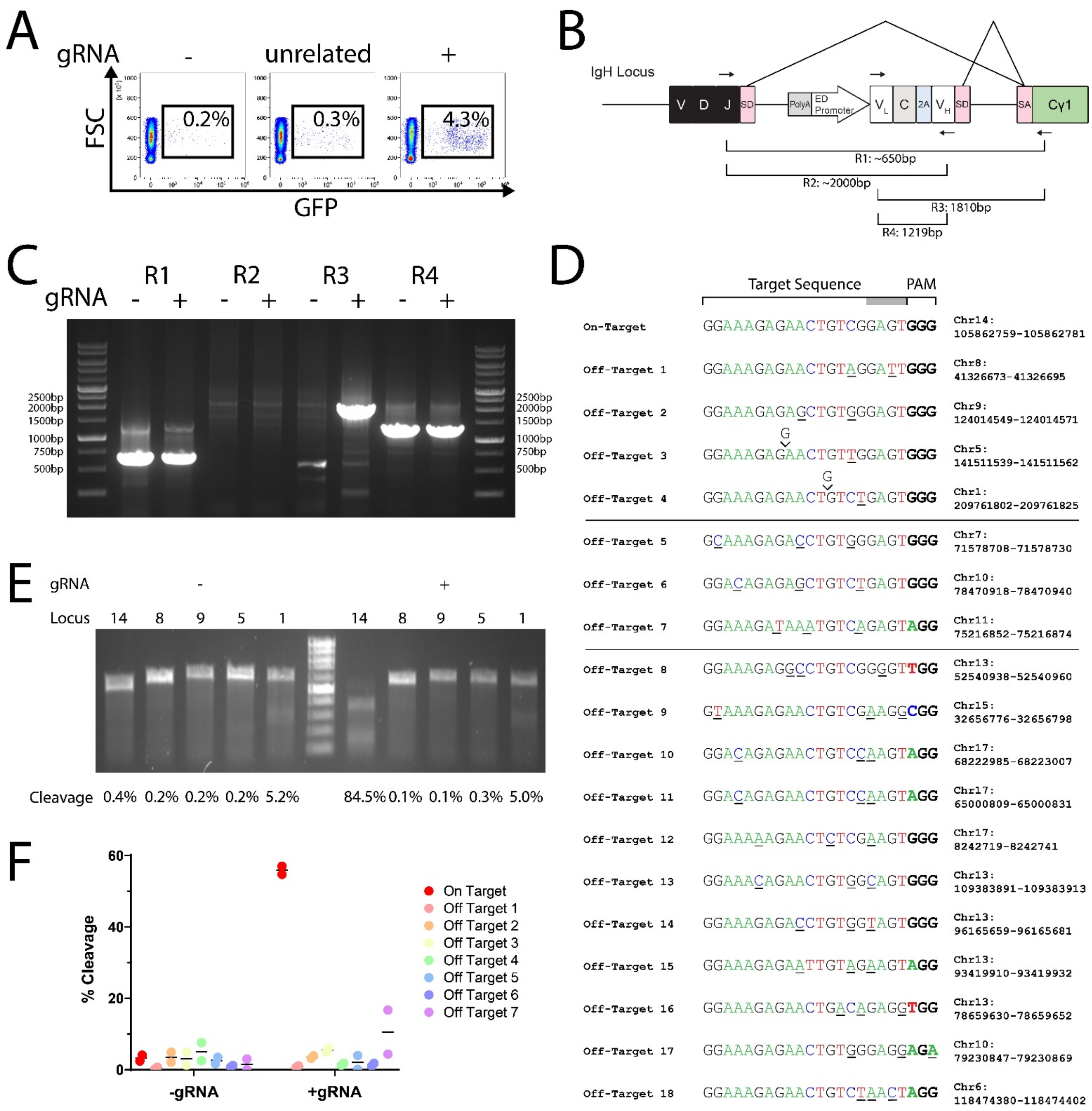

G

Mismatch	Number of Found Targets
4	135
5	1457
6	11087

H

Mismatch	Number of Found Targets
0	1
1	0
2	0
3	6
4	188
5	1988
6	13514

Supplementary Figure 4. (A) Flow cytometry plots demonstrating CRISPR/Cas9-dependent integration of a GFP cassette, as in S2C, under an enhancer-dependent promoter into the IgH locus of murine splenic B cells. Gating on live, singlets. **(B)** Scheme of the IgH locus following engineering. Primers for amplification are annotated as arrows. Primer pairs for each reaction (R1 to 4) and respective expected amplicon sizes are indicated. Cones above represent expected splicing. **(C)** RTPCR amplification of RNA from cells engineered with (+) or without (-) the specific gRNA. Primer pairs used as indicated in (B). Ladder size indicated on each side of the gel **(D)** Depicted are all potential off-target sites for the human IgH gRNA that were detected using COSMID[35] to have no more than 2 differences (1-4), or with 3 mismatches and identical 4 PAM proximal positions (5-7) or with three mismatches at any position (9-18), with respect to the desired on-target site on chromosome 14 (top). Chromosomal locations are indicated on the right. Cones above the sequence indicate nucleotide insertions. Underlined nucleotides indicate mismatches. **(E)** T7E1 cleavage assay using DNA amplified from on-target and off-target sites, in cells transfected either with (+) or without (-) the specific human IgH gRNA. chromosomal location, as in (D), is indicated above. Calculated efficiency is indicated below. **(F)** TIDE analysis of Sanger sequencing from the 7 first off target sites as in (D). Each dot represents a separate TIDE assay, either comparing two –gRNA samples (-gRNA) or Cas9 and gRNA electroporated cells compared to a –gRNA sample (+gRNA). **(G)** Table indicating possible off target sites for the human gRNA with 4 to 6 mismatches. **(H)** Table indicating possible off target sites for the murine gRNA. For 1-2 mismatches, no off-target sites were detected.

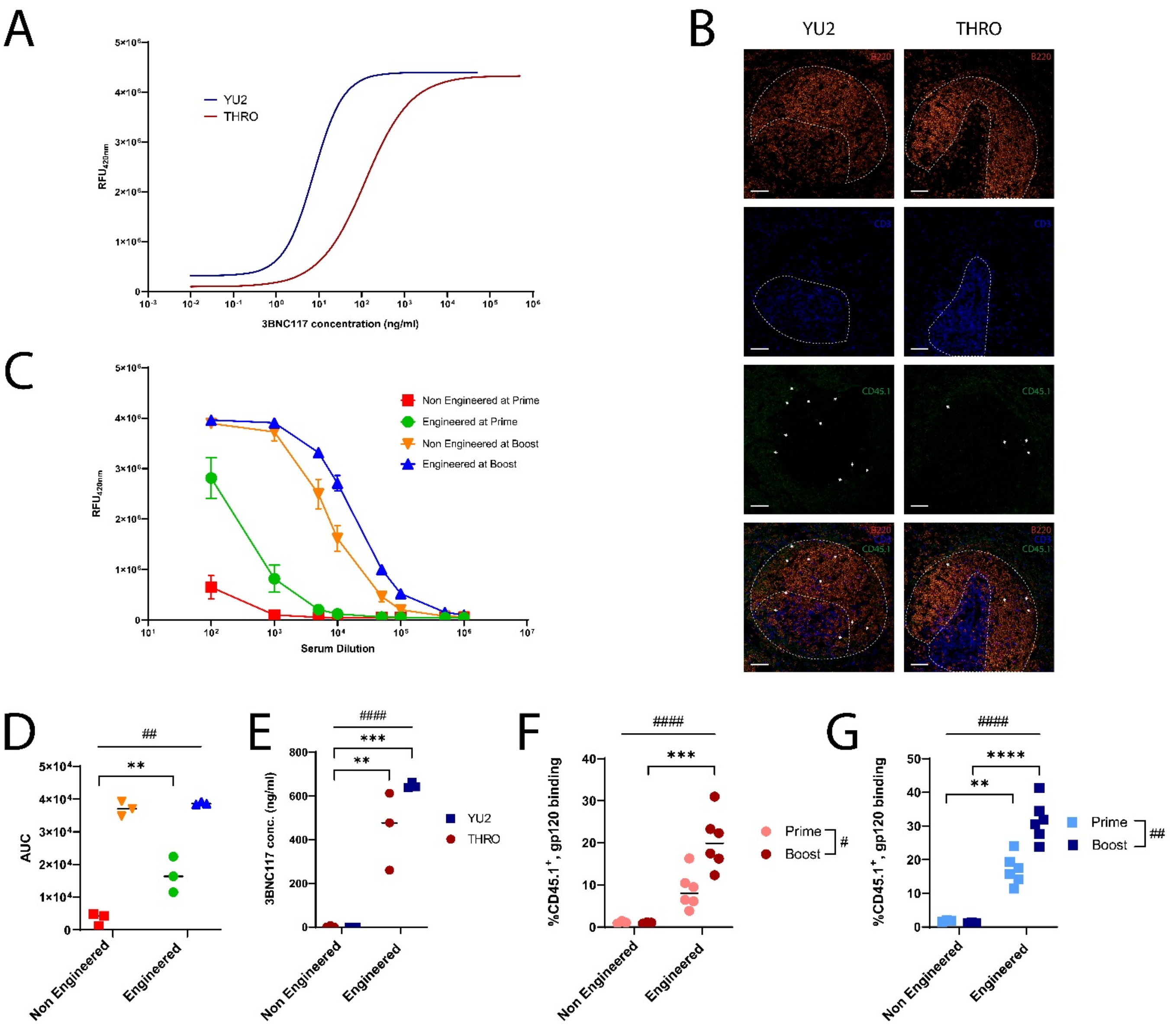

Supplementary Figure 5. (A) Analysis by ELISA for the binding of 3BNC117 to either the YU2.DG (Blue) or the THRO4156.18 (Orange) gp120 antigen. **(B)** Histologic sections of spleens from CD45.2 mice receiving adoptive transfer of engineered CD45.1 cells. Arrows indicate CD45.1+ B220+ cells. Contour indicates the dark and light zones in the germinal centers. Bar on the bottom left represents a relative scale unit. For each antigen, the experiment was reproduced twice, independently. **(C)** ELISA specific for the YU2.DG gp120 antigen performed on sera from mice immunized with the YU2.DG gp120 antigen. (n=3, each dot represents the mean value of independent animals, +/- SD). **(D)** Quantification of (C) using Area under the Curve (AUC). ##pv=0.0031 for Two-way ANOVA and **pv=0.0035 (n=3, each dot represents an independent animal). **(E)** ELISA of sera from mice receiving engineered or non-engineered cells. The sera were collected 14 days after boost immunizations with either the YU2.DG or the THRO. 4156.18 gp120 antigens. Quantification using an anti-idiotypic antibody to 3BNC117. ####pv<0.0001; Two-way ANOVA and ***pv=0.0001 and **pv=0.0016 for Tukey's multiple comparison (n=3, each dot represents an independent animal). **(F)** and **(G)** Quantification of analysis by flow cytometry of CD45.1 expression and gp120 binding in the GCs of mice following prime or boost immunizations, gating on live, singlets, $B220^{+}$, $GL\text{-}7^{+}$. Non-engineered: mice receiving adoptive transfer of non-engineered CD45.1 cells as in (3A). Mice were immunized with either the THRO4156.18 (F) or YU2.DG (G) gp120 antigen.

####pv<0.0001, ##pv=0.0071, #pv=0.0281 for two-way ANOVA and ****pv<0.0001 ***pv=0.0002 and **pv=0.0013 for Tukey's multiple comparison (n=3 for the Non Engineered and n=6 for the Engineered groups, each dot represents a biologically independent animal).

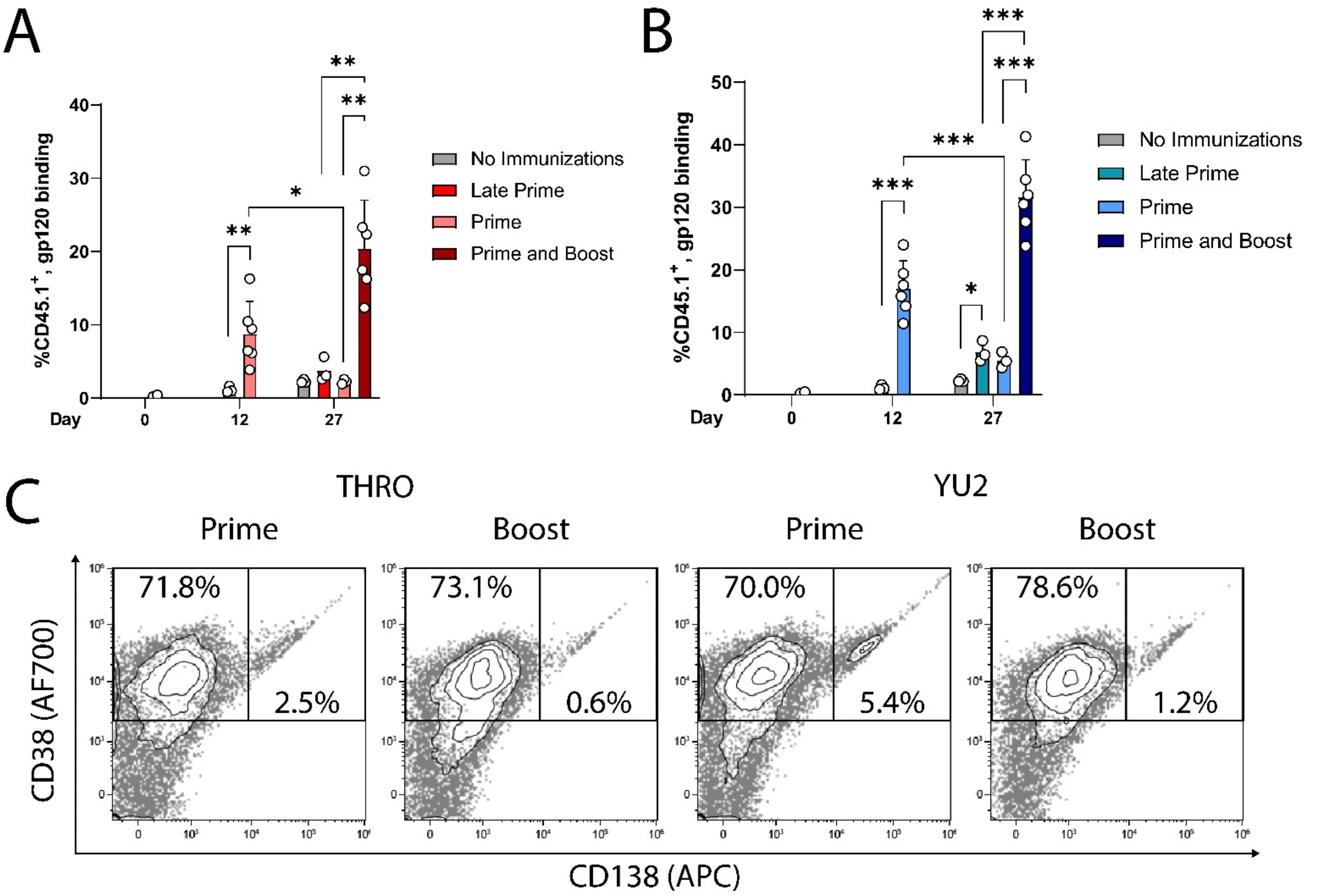

Supplementary Figure 6. (A-B) Analysis by flow cytometry, as in Fig. 4B, for immunizations with either the THRO4156.18 (A) or the YU2.DG (B) gp120 antigens, with the addition of the mice cohorts: "late prime", "no boost" and "no immunization" (adjuvant only). Gating on live, singlets, B220^{+}, GL-7^{+}. P values indicated from left to right (A): **pv=0.0077, *pv=0.0146, **pv=0.004, **pv=0.001. P values indicated from left to right (B): ***pv=0.0003, ***pv=0.0008, *pv=0.0389, ***pv=0.0003, ***pv=0.0002 for two-tailed t-test (n=2 for day 0, n=3 for the No Immunizations groups, n=3 for the Prime and Late Prime groups at day 27, n=6 for the Prime at day 12 and Prime and Boost at day27 groups, each dot represents an independent animal, data represented as mean values +/- SD). **(C)** Representative flow cytometry for Fig.4D-E. Gated on live, singlets, CD45.1+.

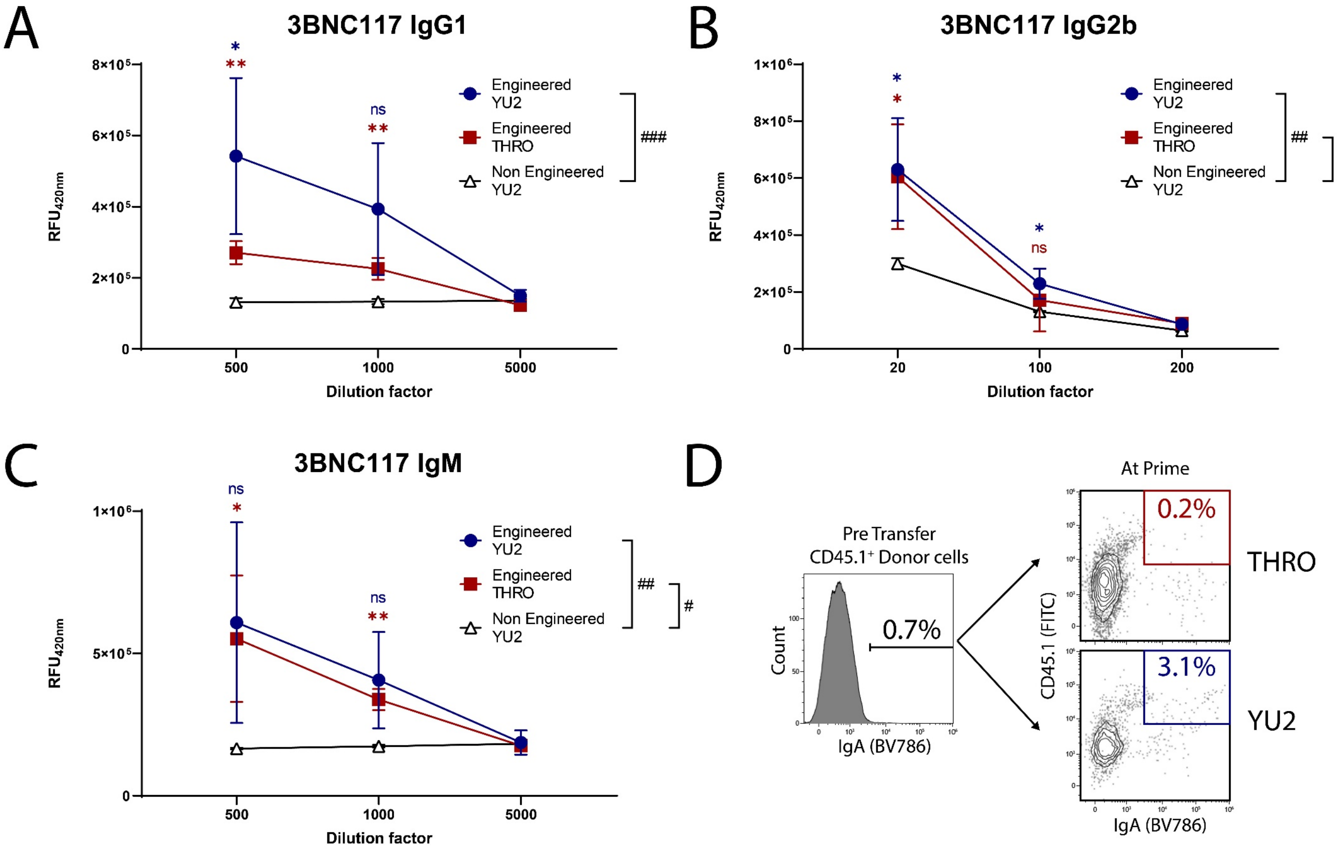

Supplementary Figure 7. (A-C) Isotype specific anti-idiotypic ELISA measuring 3BNC117 isotypes in mice sera collected after boost immunizations. ###pv=0.0002, ##(B) = pv=0.0068, #(B) = pv=0.0242, ##(C) = pv=0.0098, #(C) = pv=0.0376, for Dunnett's multiple comparisons and, from left to right, top to bottom: *pv=0.0318, **pv=0.0021, **pv=0.0071, *pv=0.0343, *pv=0.0461, *pv=0.0321, *pv=0.0397, **pv=0.0016 for two-tailed t-test on single dilution comparisons. Comparisons performed to sera of mice receiving adoptive transfer of non-engineered B cells and boost immunized with YU2.DG (n=3, data represented as the mean value of biologically independent animals, +/-SD). **(D)** Representative flow cytometry of IgA expression before (out of CD19+, live, singlets) or after transfer and prime immunization (out of live, singlets, GL-7+, B220+)

A

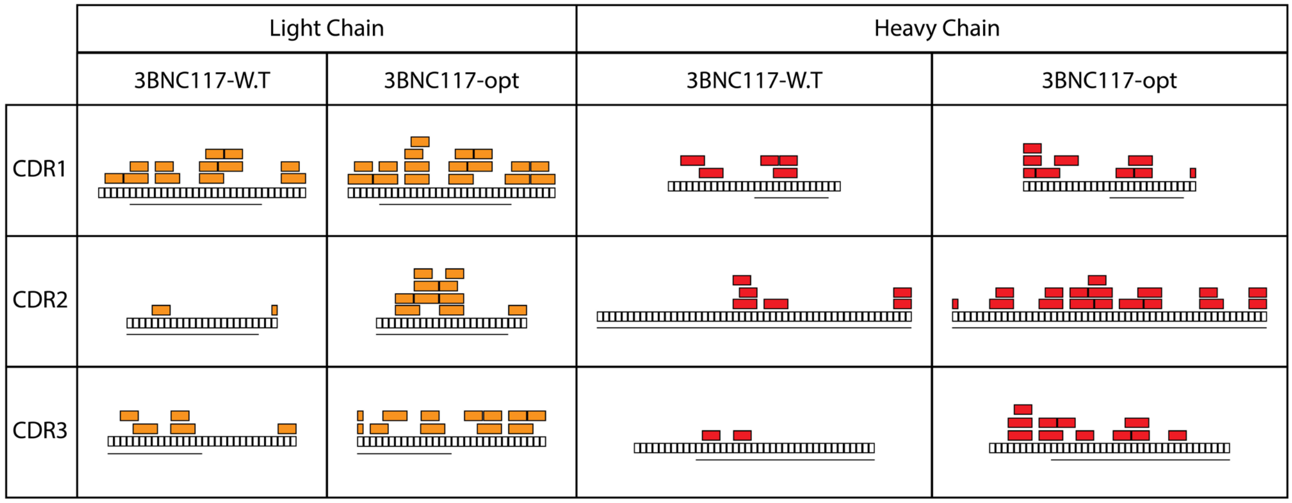

B

Sequence	Hotspot	V_H	V_L
**3BNC117-W.T.**	WRCH/DGYW	**16**	**19**
	RCY/RGY	**45**	**28**
	C/G	**204**	**144**
**3BNC117-opt.**	WRCH/DGYW	**38**	**36**
	RCY/RGY	**65**	**52**
	C/G	**220**	**170**

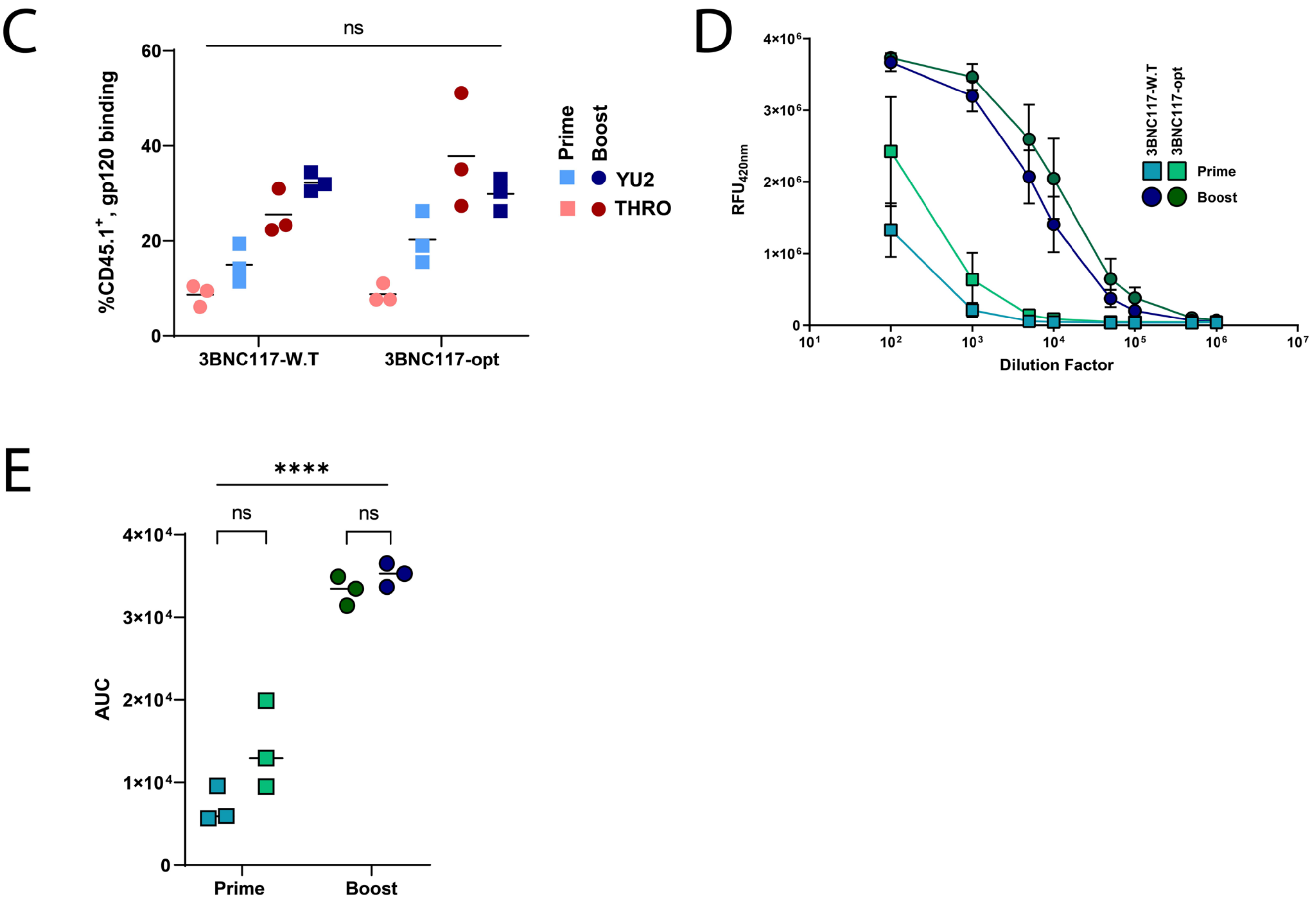

Supplementary Figure 8. (A) Synonymous re-coding of 3BNC117 to include more AID hotspots for enhanced SHM potential. The scheme depicts the loci surrounding the CDR loops for the Kappa Light Chain and Heavy Chain of 3BNC117. Each box represents a base pair. Bars below indicate the CDR loops. Colors, either yellow for the light chain or red for the heavy chain, represent hotspots (either RCY or WRCH). Overlapping hotspots are stacked **(B)** Table summarizing the quantity of hotspots in the 3BNC117-W.T. and 3BNC117-opt. donors. **(C)** Analysis by flow cytometry of CD45.1 expression and gp120 binding in the GCs of mice following prime or boost immunizations of mice receiving B cells engineered with either 3BNC117-W.T. or 3BNC117-opt, gating on live, singlets, B220+, GL-7+. Three-way ANOVA for comparison of mice receiving adoptive transfer of B cells engineered with either the W.T. or 3BNC117-opt coding vectors (n=3, each dot represents a biologically independent animal). **(D)** ELISA specific for the YU2.DG gp120 antigen performed on sera from mice immunized with the YU2.DG gp120 antigen (n=3, data represented as the mean value of biologically independent animals, +/-SD). **(E)** Area under the curve (AUC) quantification of (D). ****pv < 0.0001, Two-way ANOVA (n=3, each dot represents a biologically independent animal).

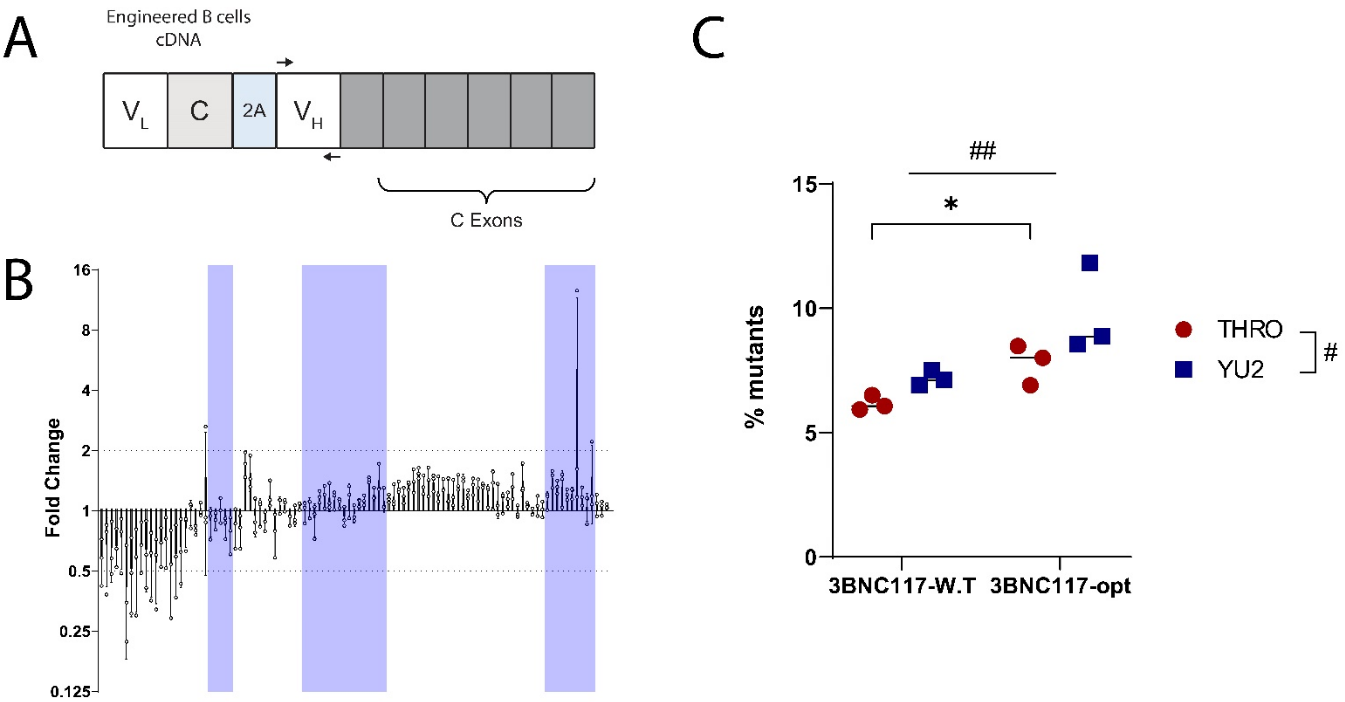

Supplementary Figure 9. (A) Scheme of cDNA amplification for Illumina sequencing from splenic lymphocytes. Primers are depicted as arrows. **(B)** Mutational landscape in the 3BNC117 V_H chain of mice, boost immunized twice with the YU2.DG gp120 antigen (n=3) compared to that of the engineered cells before adoptive transfer. Each bar represents a base pair on the V_H. For each position, the frequency of mutation is compared before adoptive transfer and after boost immunization. Blue shading indicates CDR loops (n-=3, each dot represents a biologically independent animal, data represented as mean values +/-SD). **(C)** Fraction of the mutant clones, from mice receiving adoptive transfer of either 3BNC117-W.T. or 3BNC117-opt engineered cells. ##pv=0.0071, #pv=0.035; Two-way ANOVA and *pv=0.0108 two-tailed t-test (n=3, each dot represents a biologically independent animal).

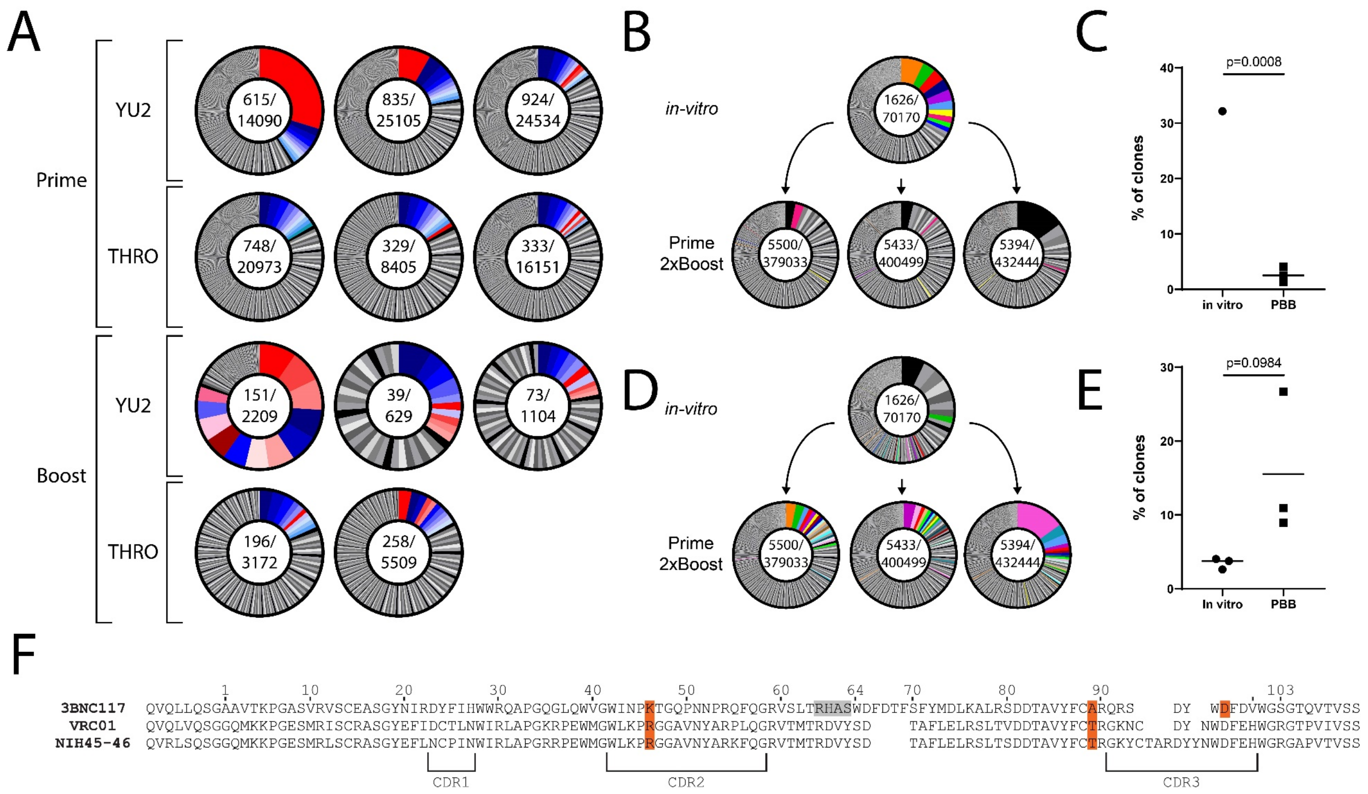

Supplementary Figure 10. Antigen induced clonal expansion. **(A)** Pie charts of 3BNC117 mutant clones from the spleens of mice immunized with the gp120 antigen of the YU2.DG or THRO.4156.18 strains. Each pie represents a mouse.10-12 most abundant clones were colored. Shades of red indicate clones that were not found in the most abundant clones of other mice. Shades of blue indicate shared clones. Indicated numbers represent number of different mutant clones out of the total number of reads **(B-E)** Engineered cells of the same batch, before and after adoptive transfer and immunizations, were analyzed for the presence of similar clones **(B)** Here, the color code is used to follow the distribution of the 10 most abundant pre-adoptive-transfer clones. Indicated numbers represent number of different mutant clones on total number of reads **(C)** Quantitation of (B) by summing the frequency of the 10 clones. PBB = Prime immunization followed by two boost immunizations. p value as indicated for a one sample t-test (n=3, each dot represents the frequency of the 10 most abundant clones prior to adoptive transfer compared to the same clone's frequency after adoptive transfer and immunizations). **(D)** Here, the color code is used to follow the distribution of the 10 most abundant clones in each mouse. Indicated numbers represent number of different mutant clones out of the total number of reads. **(E)** Quantitation of (D). PBB = Prime immunization followed by two boost immunizations. p value as indicated for two-tailed t-test (n=3, for the in-vitro and PBB, each dot represents the frequency of the 10 most abundant clones from biologically independent animals compared to the same clone's frequency prior to adoptive transfer). **(F)** Alignment of the V_H sequences of the 3BNC117, the VRC01 and the NIH45-46 bNAbs. The CDRs of 3BNC117 are annotated in the figure. Orange colored bases represent sites of mutations allowing for clonal expansion reaching above 10% of the mutant sequences, in any given mouse. Converging substitutions between VRC01/NIH45-46 and 3BC117 mutants from mice are colored.

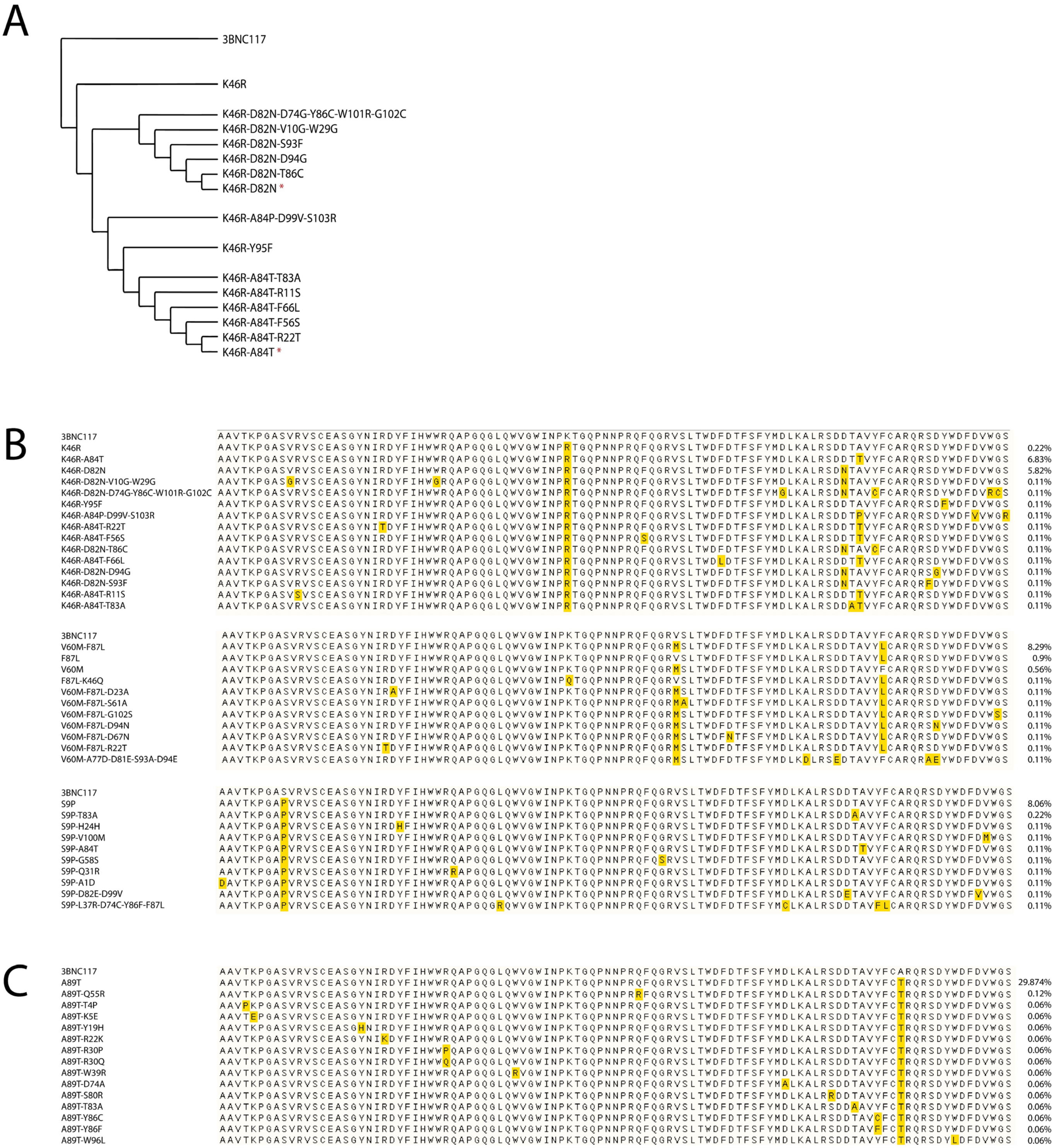

Supplementary Figure 11. Distribution of specific mutations in immunized mice indicates Somatic Hypermutation and clonal expansion **(A)** Lineage reconstruction from the K46R mutation in a gp120 YU2.DG immunized mouse implies SHM and clonal expansion (Fig.5E, S10A). **(B)** K46R, V60M and S9P lineages in the same mouse as (B) aligned to the original 3BNC117 sequence (top). Numbers on the right represent the frequency of each clone. **(C)** Same as (B) but for the A89T lineage in a different mouse.

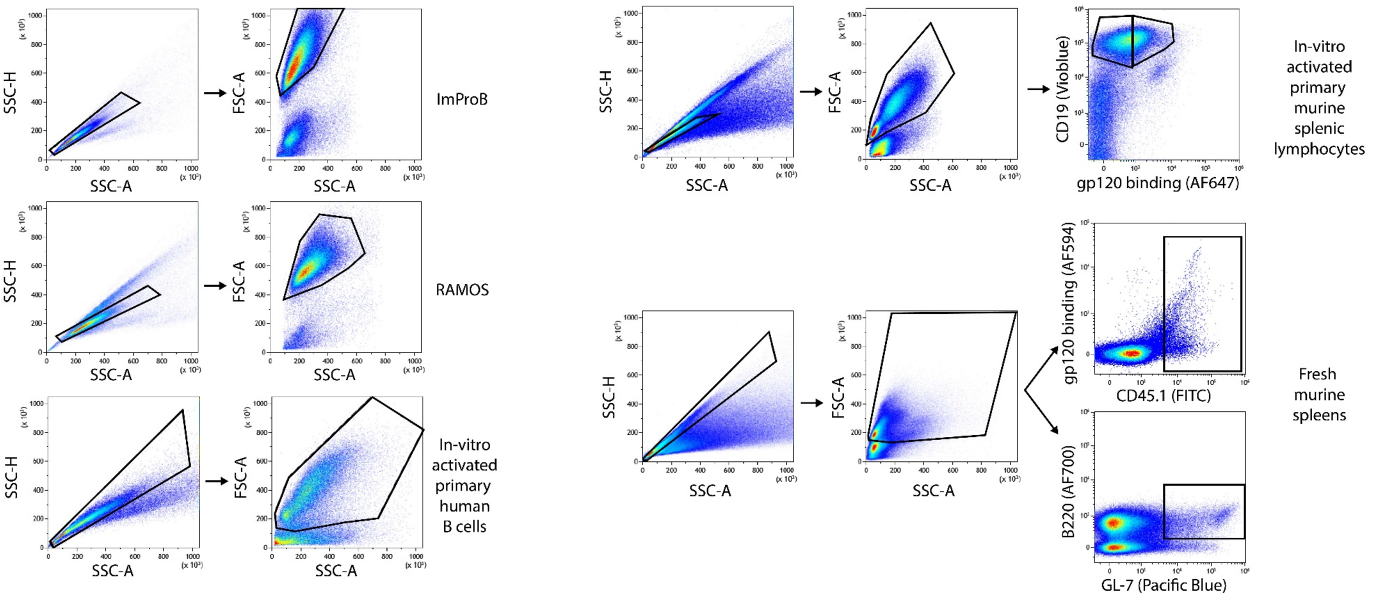

Supplementary Figure 12. Representative gating strategy for the different experiments.

SUPPLEMENTARY TABLES:

Supplementary Table 1:

List of gRNA used in this manuscript	
Mouse IgH	CGAUGCAUAGGGACAAAGAG
Mouse IgK	UGGUGCAGCAUCAGCCCCUG
Human IgH	GGAAAGAGAACUGUCGGAGU
Human IgK	UGGUGCAGCCACAGUUCCUG
Mouse “unrelated” control	CAGACCCAACAAGAAGACCA

Supplementary Table 2

List of antibodies used in this manuscript					
Name	Clone	Label	Source	Cat #	Dilution
Anti-mouse CD45.1	A20	FITC	Invitrogen	10-0453-82	1:100
Anti-mouse IgG1	A85-1	BV421	BD Bioscience	562580	1:100
Anti-mouse IgA	C10-1	BV786	BD Bioscience	743298	1:100
Anti-mouse CD19	6D5	BV421	Biolegend	115537	1:100
Anti-mouse CD19	6D5	FITC	Biolegend	152403	1:100
Anti-mouse CD45RA/B220	1D3/CD19	AF700	Biolegend	103232	1:100
Anti-mouse CD138	281-1	APC	Biolegend	142506	1:100
Anti-mouse GL7 antigen	GL-7	PerCP CY5.5	Biolegend	144609	1:100
Anti-mouse GL7 antigen	GL-7	Pacific blue	Biolegend	144613	1:100
Anti-mouse CD80	16-10A1	PE	Biolegend	104707	1:100
Anti-mouse/Human pERK1/2	MILAN8R	PE	eBioscience	12-9109-41	1:100
Anti-mouse CD38	90	AF700	eBioscience	56-0381-82	1:100
Anti-His-tag	OGHis	AF488	MBL	D291-A48	1:100
Anti-His-tag	OGHis	AF594	MBL	D291-A59	1:100
Anti-His-tag	6-His	FITC	Biolegend	906105	1:100
Anti-His-tag	6-His	AF647	Biolegend	906116	1:100
Anti-mouse IgK	187.1	Vioblue	MACS	130-105-860	1:100
Anti-human IgK	MHK-49	BV421	Biolegend	392705	1:100
Anti-human IgG Fc	Polyclonal	PE	eBioscience	12-4998-82	1:100
Anti-3BNC117	4E4		Produced in-house	Produced in-house	5ug/ml
Anti-mouse IgM	Polyclonal	HRP	Jackson Immunoresearch	715-035-140	2ug/ml
Anti-mouse IgG	Polyclonal	HRP	Jackson Immunoresearch	715-035-151	2ug/ml
Anti-mouse IgG1	Polyclonal	HRP	Jackson Immunoresearch	115-035-205	2ug/ml
Anti-mouse IgA	Polyclonal	HRP	abcam	ab97235	
Anti-mouse IgA	Polyclonal	HRP	SouthernBiotech	1040-05	2ug/ml
Anti-mouse IgG2b	Polyclonal	HRP	BioRad	STAR-134P	2ug/ml
Anti-mouse IgG2c	Polyclonal	HRP	BioRad	STAR-135P	2ug/ml

Supplementary Table 3:

Primers used for PCR amplification as in Fig. S2E :	
Forward	CGCGCGAAACGCGGAAG
Reverse (IgG2A)	CCACCACAGAGGAGAAGATCCAC
Reverse (IgM)	CGTGGTGGGACGAACACATTTAC
Reverse (IgA)	CATGTGAGGCTGGCATCTGAAC

Supplementary Table 4:

Primers used for PCR amplification for gRNA activity	
	Murine IgH
Forward	GGATATTTGTCCCTGAGGGAGCC
Reverse	GCCATCTTGACTCCAACTCAACATTG
	Murine IgK
Forward	AGTCCAACTGTTCAGGACGCC
Reverse	GTGTGGCTAAAAATTGTCCCATGTGG
	Human IgH
Forward	GCTGAGGAATGTGTCTCAGGAGC
Reverse	CCTCAATTCCAGACACATATCACTCATGG
	Human IgK
Forward	GCTGGAACAGTCAGAAGGTGGAG
Reverse	GCTGTCCTTGCTGTCCTGCT

Supplementary Table 5:

For assessment of HDR mediated integration, PCR was performed using primers	
Forward	CGCGCGAAACGCGGAAG
Reverse	ATATCACGCGTGTACACTAGCCAGTTTCGGCTGAATCCTCA

Supplementary Table 6:

Primers used for PCR amplification as in Fig. S4C	
Reaction 1 (R1)	mIgHJ Multiplex
	GAAAGCTGCACTGTTGACCCTG
Reaction 2 (R2)	mIgHJ Multiplex
	CACTCCACTCCTCGAGGACTCACCTGACGAGACAGT
Reaction 3 (R3)	ATCTAGAATGGACATGAGGGTCCCTGC
	GAAAGCTGCACTGTTGACCCTG
Reaction 4 (R4)	ATCTAGAATGGACATGAGGGTCCCTGC
	CACTCCACTCCTCGAGGACTCACCTGACGAGACAGT
mIgHJ Multiplex Primers:	GCACAGGGACCACGGTCAC
	GCCAAGGCACCACTCTCACAG
	GACTCTGGTCACTGTCTCTGCAG
	GGTCAAGGAACCTCAGTCACCG

Supplementary Table 7:

Primers used for PCR amplification as in Fig. S4E-F	
Off Target site	Primer Sequence
Off Target 1	CTCCTCTGTTGCCCAGACTG
	GGAAGTCCTCCTTTGTCTGATGTTCC
Off Target 2	GCCACCACAAGTTGGGTGATAAC
	GAGACTTCTACTTCACCCAGAGTCTC
Off Target 3	AAACAGAAGACTGGTCCCCAGG
	CCTCAGAGTTTCAGGGCTAACC
Off Target 4	GAAATGATCCTAGAGCCCAGTTGC
	CATGGGTTTAGAACTCCAGACTCC
Off Target 5	CCAACTCCTAGAATCAGGTGATCC
	CTCTGGGATGGACCATGATGC
Off Target 6	ATGATGGTTGAAGGAGAGCCAGTG
	TGAGAACCCAAGGCCTTCAGAATG
Off Target 7	AAGTGGGAGCTGGACAATGAGAAC
	GGGGACACCATAGCAATGCAC

Supplementary Table 8:

<table>
<tr><td colspan="3">Illumina Sequencing Primers</td></tr>
<tr><td colspan="2">Amplification of the 3BNC117-W.T VH fragment was performed using primers</td><td rowspan="6">X stands for 0-7 degenerate positions (“N”s)</td></tr>
<tr><td>Forward</td><td>TCGTCGGCAGCGTCAGATGTGTATAAGAGACAGXCAGGTCCAATTGTTACAGTCTG</td></tr>
<tr><td>Reverse</td><td>GTCTCGTGGGCTCGGAGATGTGTATAAGAGACAGXCTGACGAGACAGTGACCTGGGT</td></tr>
<tr><td colspan="2">Amplification of the 3BNC117-opt VH fragment was performed using primers</td></tr>
<tr><td>Forward</td><td>TCGTCGGCAGCGTCAGATGTGTATAAGAGACAGXCAGGTTCAGCTGCTGCAATCTG</td></tr>
<tr><td>Reverse</td><td>GTCTCGTGGGCTCGGAGATGTGTATAAGAGACAGXCTGACGAAACAGTAACTTGGGTTCC</td></tr>
<tr><td>Forward</td><td>AAGCAGAAGACGGCATACGAGATYGTCTCGTGGGCTCGG</td><td rowspan="2">Y stands for i7 indexes</td></tr>
<tr><td>Reverse</td><td>AATGATACGGCGACCACCGAGATCTACACTAGATCGCTCGTCGGCAGCGTC</td></tr>
</table>
