# Supplementary material for: Engineered B cells expressing an anti-HIV antibody enable memory retention, isotype switching and clonal expansion
Source: Nat Commun. 2020 Nov 17;11:5851. doi: 10.1038/s41467-020-19649-1 (PMC7673991; doi:10.1038/s41467-020-19649-1)
Supplement: Supplementary file 2 — Descriptions of Additional Supplementary Files [file 41467_2020_19649_MOESM2_ESM.pdf]

Descriptions of Additional Supplementary Files

Supplementary Data 1

Description: Alternative target sites in the IgHJ-iEmu intron

Supplementary Data 2

Description: Primers used for PCR amplification of the murine homology arm
